# OTUD6A in Airway Epithelial Cells Exacerbates Allergic Asthma by Promoting Airway Inflammation and Airway Remodeling Through Deubiquitination of hResistin/mRELMα

**DOI:** 10.1002/advs.202516355

**Published:** 2026-01-20

**Authors:** Weiting Pan, Xinru Xi, Wei Dai, Tingfang Xiao, Yeqing Chen, Xuanyu Chen, Xiangting Ge, Chengguang Zhao, Hui Zhang, Yali Zhang, Weixi Zhang

**Affiliations:** ^1^ Department of Pediatric Allergy and Immunology The Second Affiliated Hospital and Yuying Children's Hospital of Wenzhou Medical University Wenzhou Zhejiang China; ^2^ Chemical Biology Research Center School of Pharmaceutical Sciences Wenzhou Medical University Wenzhou Zhejiang China; ^3^ Zhejiang Provincial Clinical Research Center For Pediatric Disease Wenzhou Zhejiang China

**Keywords:** airway epithelial cells, airway inflammation, airway remodeling, asthma, ovarian tumor deubiquitinase 6A

## Abstract

Asthma is a prevalent chronic airway disorder characterized by airway hyperresponsiveness (AHR), persistent airway inflammation, and airway remodeling. Emerging evidence implicates ubiquitination‐a critical post‐translational modification‐in asthma pathogenesis. The study identifies ovarian tumor deubiquitinase 6A (OTUD6A), a deubiquitinase with established oncogenic roles, as a novel regulator of airway inflammation and remodeling. The study finds a significantly upregulation of OTUD6A in asthma patients and murine lungs, with predominant localization in airway epithelial cells. Genetic ablation of Otud6a prevents house dust mite (HDM)‐induced AHR, airway inflammation, mucin hypersecretion in both chronic and acute asthma models, as well as airway remodeling in chronic asthma model. Mechanistically, multi‐omics analysis identifies the secreted cytokine human (h)Resistin/mouse resistin‐like molecule α (mRELMα) as a substrate of OTUD6A. OTUD6A deubiquitinates and stabilizes hResistin by specifically removing its K48‐linked polyubiquitin chains at lysines K2 and K19 via the catalytic residue C152, thereby blocking its proteasomal degradation and promoting its secretion. The consequent accumulation of hResistin potentiates epithelial alarms production and facilitates epithelial‐mesenchymal transition, driving airway inflammation and airway remodeling. Furthermore, adeno‐associated virus 6‐mediated OTUD6A silencing in murine lungs markedly ameliorates asthma phenotypes. These findings establish a pathogenic OTUD6A‐hResistin/mRELMα axis and nominating OTUD6A as a promising therapeutic target for asthma intervention.

## Graphical abstract

This study elucidates a novel mechanistic role of the deubiquitinase OTUD6A in asthma pathogenesis, uncovering its regulatory function in airway inflammation and airway remodeling through the stabilization of hResistin/mRELMα. This study offers a novel regulatory axis (OTUD6A‐hResistin/mRELMα) in asthma pathogenesis and OTUD6A inhibition as a potential therapeutic strategy.

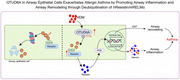

**Abbreviations**
3‐MA: 3‐methyladenine
AAV6: adeno‐associated virus serotype 6
AECs: airway epithelial cells
AHR: airway hyperresponsiveness
ALT: alternaria alternate
Ang II: angiotensin II
BALF: bronchoalveolar lavage fluid
BRD4: bromodomain‐containing protein 4
BSA: bovine serum albumin; BTZ, Bortezomib
cDNA: complementary DNA
CHX: cycloheximide
Co‐IP: co‐immunoprecipitation
CQ: chloroquine
DAB: 3,3‐diaminobenzidine
DAMP‐RAGE: damage‐associated molecular patterns‐receptor for advanced glycation end‐products
DUBs: deubiquitinating enzymes
EMT: epithelial‐mesenchymal transition
ER: endoplasmic reticulum
ERAD: ER‐associated degradation
Ers: expiratory reserve volume
FDR: false discovery rate
H&E: hematoxylin and eosin
HDM: house dust mites
HDM‐AAM: HDM‐induced acute asthma model
HDM‐CAM: HDM‐induced chronic asthma model
HMGB1: high mobility group box‐1
HRP: horseradish peroxidase
IL: interleukin
ITGB4: integrin beta 4
LC‐MS/MS: liquid chromatography‐tandem mass spectrometry
mRELMα: mouse resistin‐like molecule α
NLRP3: nucleotide‐binding oligomerization domain‐like receptor family pyrin domain containing 3
OTUD6A: ovarian tumor deubiquitinase 6A
OVA: ovalbumin
PAS: periodic acid‐Schiff
PBS: phosphate‐buffered saline
PI3K/AKT: phosphatidylinositol 3 kinase/ protein kinase B
PTMs: post‐translational modifications
Rn: airway resistance
RNA‐seq: RNA sequencing
Rrs: respiratory rate signal
RT‐qPCR: real‐time quantitative polymerase chain reaction
SEM: standard error of the mean
SHP2/JNK/c‐Jun/FGF2: Src homology domain 2‐containing protein tyrosine phosphatase 2/c‐Jun N‐terminal kinase/Jun N‐terminal kinase‐dependent transcription factor/fibroblast frowth factor 2
STAT3: signal transducer and activator of transcription 3
STING: stimulator of interferon genes
TGFβ1: ransforming growth factor β1
Th2: T helper 2
TMB: tetramethylbenzidine
TMT: tandem mass tag
TNFAIP3: tumor necrosis factor, alpha‐induced protein 3
TSLP: thymic stromal lymphopoietin
UBE2N: ubiquitin conjugating enzyme E2 N
USPs: ubiquitin‐specific peptidases
WT: wild‐type
ZO‐1: zonula occludens‐1

## 1 Introduction

Asthma is a prevalent chronic inflammatory airway disease involving multiple cell types and is one of the most significant chronic conditions affecting global health [1]. Its primary pathological features include airway hyperresponsiveness (AHR), chronic airway inflammation, and airway remodeling [1, 2]. Allergic asthma, the most common asthma phenotype, is predominantly triggered by inhaled allergens such as house dust mites (HDM), pollen, and fungal spores. Airway epithelial cells (AECs), acting as the first line of defense, play a pivotal role in allergen recognition and signal transduction [3, 4]. Upon allergen exposure, these cells activate key intracellular regulatory proteins, leading to cellular damage and subsequent release of alarmins (e.g., interleukin (IL)‐25, IL‐33, thymic stromal lymphopoietin (TSLP)) or initiation of epithelial‐mesenchymal transition (EMT), which drive airway inflammation and remodeling, respectively [5]. Despite widespread emphasis on standardized diagnosis and treatment, current clinical therapies‐primarily combining glucocorticoids with β_2_‐adrenergic receptor agonists‐only provide symptomatic control rather than a cure [6]. Thus, identifying critical regulatory proteins within AECs may represent a promising therapeutic strategy for allergic asthma prevention and treatment.

Post‐translational modifications (PTMs) are critical steps in protein biosynthesis, involving chemical modifications that alter protein function [7]. Among these, ubiquitination refers to the covalent attachment of ubiquitin moieties to substrate proteins via the action of ubiquitin enzymes. The ubiquitin‐proteasome pathway serves as a predominant mechanism for endogenous protein degradation and plays a central role in selective intracellular protein turnover. Ubiquitination is a dynamic process that can be reversed by deubiquitinating enzymes (DUBs). Emerging evidence implicates several DUB family members‐including A20/tumor necrosis factor, alpha‐induced protein 3 (TNFAIP3), ubiquitin‐specific peptidases (USPs) 4, 7, 21, and 38‐in the pathogenesis of allergic asthma [8, 9, 10, 11, 12]. Ovarian tumor deubiquitinase 6A (OTUD6A), a member of the OTU‐family DUBs, has been demonstrated to regulate cell proliferation and tumorigenesis [13, 14]. OTUD6A also mediates inflammatory and pathological processes by deubiquitinating key signaling proteins, such as nucleotide‐binding oligomerization domain‐like receptor family pyrin domain containing 3 (NLRP3), ubiquitin conjugating enzyme E2 N (UBE2N/UBC13), signal transducer and activator of transcription 3 (STAT3), and stimulator of interferon genes (STING), to mediates inflammatory bowel disease/colitis, viral infection, angiotensin II (Ang II)‐induced renal injury and pathological cardiac hypertrophy, respectively [15, 16, 17, 18]. However, whether OTUD6A contributes to allergic asthma remains unexplored, highlighting a gap in understanding its role in type 2 inflammation and airway remodeling.

This study bridges this gap by defining OTID6A as a master regulator of allergic asthma. We demonstrate that OTUD6A expression is markedly upregulated in AECs both in acute and chronic murine models of allergic asthma. Genetic ablation or lung‐specific knockdown of OTUD6A conferred robust protection against AHR, airway inflammation, and airway remodeling. Mechanistically, we identified human (h)Resistin/mouse resistin‐like molecule α (mRELMα) as the target substrate of OTUD6A. OTUD6A mediates the expression of epithelial‐derived alarms and EMT via deubiquitinating and stabilizing hResistin/mRELMα. Our findings establish OTUD6A as a master regulator of airway inflammation and remodeling via hResistin/mRELMα, nominating it as a tractable therapeutic target for allergic asthma.

## 2 Results

### 2.1 AECs‐Derived OTUD6A Is Markedly Upregulated in Asthmatic Lungs

**FIGURE 1:**
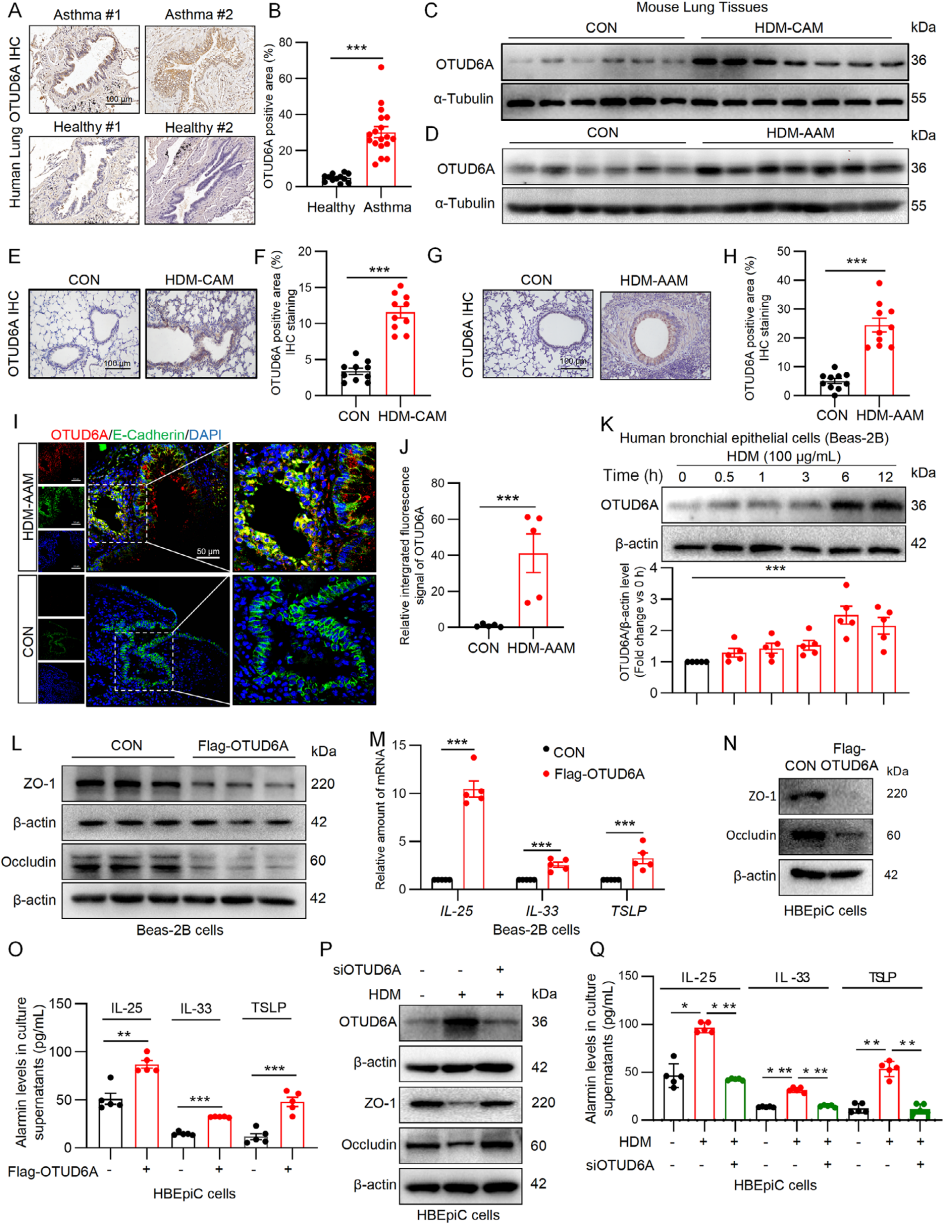
OTUD6A expression is upregulated in airway epithelial cells in asthma. A‐B) Immunohistochemical (IHC) staining and quantification of OTUD6A in human lung tissues from healthy controls (*n* = 2) and asthmatic patients (*n* = 2). Scale bars: 100 µm. C‐D) Western blot analysis of OTUD6A expression in lung tissue from HDM‐CAM (C) and HDM‐AAM (D) mice. E‐F) Representative images of IHC for OTUD6A on mouse lung sections of HDM‐CAM and corresponding quantitative analysis (*n* = 5). Scale bars: 100 µm. G‐H) Representative images of IHC for OTUD6A on mouse lung sections of HDM‐AAM and corresponding quantitative analysis (*n* = 5). Scale bars; 100 µm. I‐J) Immunofluorescence staining for OTUD6A in the mouse lung tissue of HDM‐AAM and corresponding quantitative analysis (*n* = 5). Scale bars: 50 µm. K) Western blot analysis of OTUD6A protein levels in BEAS‐2B cells stimulated with HDM (100 µg/mL) at indicated time points (*n* = 5). L‐M) BEAS‐2B cells transfected with OTUD6A or control vector for 24 h. L) Western blot analysis of ZO‐1 and Occludin. M) RT‐qPCR analysis of *IL‐25*, *IL‐33*, and *TSLP* mRNA levels (*n* = 5). N‐O) HBEpiC cells transfected with OTUD6A or control vector for 24 h. N) Western blot analysis of ZO‐1 and Occludin. O) ELISA analysis of IL‐33, IL‐25, and TSLP levels in cell supernatant (*n* = 5). P‐Q) HBEpiC cells transfected with siOTUD6A or control vector for 48 h and stimulated with HDM (100 µg/mL) for 6 h. P) Western blot analysis of OTUD6A, ZO‐1, and Occludin. Q) ELISA analysis of IL‐33, IL‐25, and TSLP levels in cell supernatant (*n* = 5). Data are presented as mean ± SEM. *P* values determined by two‐tailed unpaired t‐test or one‐way ANOVA (*p < 0.05, **p < 0.01, ***p < 0.001).

To investigate the activity of OTUD6A in asthmatic lungs, we performed immunochemistry staining on clinical specimens. Strikingly, while healthy lung tissues exhibited negligible OTUD6A‐positive signals, substantial OTUD6A immunoreactivity was detected in lung sections from asthma patients (Figure 1A,B; Figure S1A). This observation was corroborated by transcriptomic analysis of a published severe asthma dataset (GSE43696, Figure S1C). To validate these findings in experimental models, we assessed OTUD6A expression in HDM‐induced chronic asthma model (HDM‐CAM) and acute asthma model (HDM‐AAM) in mice. Western blot analysis revealed significant upregulation of OTUD6A in lung tissues of asthmatic mice compared with control groups (Figure 1C,D; Figure S1D,E). This observation was further corroborated by immunochemistry staining, which demonstrated a marked increase in OTUD6A positive foci within the pulmonary parenchyma of asthma model mice (Figure 1E–H). A similar elevation was observed in murine models of airway inflammation induced by ovalbumin (OVA), *Alternaria alternate* (Alt), and IL‐33 (Figure S1F–K). Collectively, these results indicated that OTUD6A expression is increased in the lung tissues of asthmatic patients and mice.

To identify the cellular source of up‐regulated OTUD6A in asthmatic lung, we found that OTUD6A‐positive area located in the bronchial epithelial area in the immunohistochemical pictures of lung tissues from asthmatic patients and mice (Figure 1A,E,G), so we performed double staining with OTUD6A and the AEC marker, E‐Cadherin. The results showed that HDM treatment increased OTUD6A and E‐Cadherin colocalized (Figure 1I,J), unequivocally identifying AECs as the major source of upregulated OTUD6A in asthma. Given the pivotal role of AECs in allergic asthma pathogenesis‐where allergen recognition triggers signaling cascades, disrupts intercellular junctions, and promotes cytokine release [19]‐we further investigated OTUD6A in vitro. In HDM‐stimulated human bronchial epithelial cells (BEAS‐2B), OTUD6A expression increased time‐dependently (Figure 1K). Notably, OTUD6A overexpression significantly reduced tight junction proteins (zonula occludens‐1 (ZO‐1) and Occludin, Figure 1L; Figure S1N,O) while upregulating epithelial‐derived alarms (*IL‐33*, *IL‐25*, and *TSLP*, Figure 1M). OTUD6A overexpression in human primary bronchial epithelial cells (HBEpiC) also reduced these tight junction proteins (Figure 1N; Figure S1P,Q) and epithelial‐derived alarmins (Figure 1O). Furthermore, HDM can induce OTUD6A expression in HBEpiC, and silencing OTUD6A reversed HDM‐induced decrease of ZO‐1 and Occudin (Figure 1P; Figure S1R‐T), as well as increase of epithelial‐derived alarmins levels (Figure 1Q). Therefore, these findings demonstrate that AECs‐derived OTUD6A is robustly upregulated in asthmatic lungs and may drive disease progression.

### 2.2 Otud6a Deficiency Ameliorates AHR and Airway Inflammation in HDM‐Induced Mouse Acute Asthma Model

**FIGURE 2:**
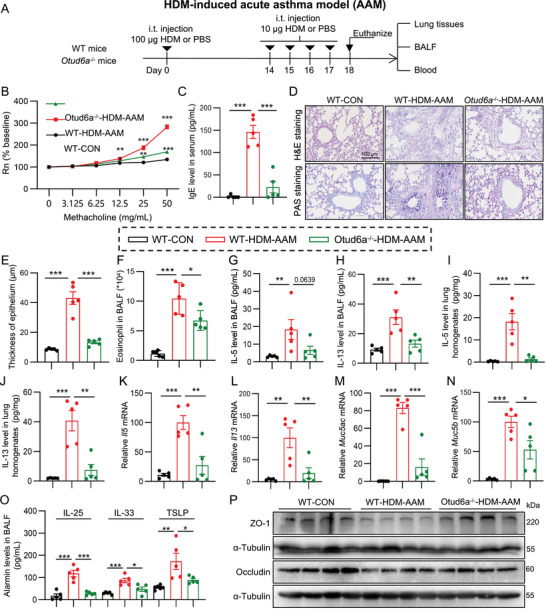
OTUD6A knockout alleviates asthma in HDM‐induced acute asthma model. A) Schematic diagram depicting the procedure of HDM‐AAM. B) AHR assessed via acetylcholine challenge (*n* = 5). C) Serum IgE levels measured by ELISA (*n* = 5). D) Representative H&E and PAS staining of lung tissue. Scale bars: 100 µm. E) Quantification of bronchial epithelial thickness (*n* = 5). F) BALF eosinophil counts determined by Wright‐Giemsa staining (*n* = 5). G‐J) IL‐5 and IL‐13 levels in BALF (G, H) and lung homogenates (I, J) measured by ELISA (*n* = 5). K‐N) RT‐qPCR analysis of *Il5*, *Il13*, *Muc5ac*, and *Muc5b* mRNA levels in lung tissue (*n* = 5). O) ELISA analysis of IL‐25, IL‐33, and TSLP levels in BALF (*n* = 5). P) Western blot and quantification of ZO‐1 and Occludin in lung tissues. Data are presented as mean ± SEM. *P* values determined by one‐way ANOVA (*p < 0.05, **p < 0.01, ***p < 0.001).

To elucidate the role of OTUD6A in asthma pathogenesis, we first assessed the impact of Otud6a knockout in HDM‐AAM (Figure 2A). Compared with wild‐type asthmatic mice, Otud6a‐deficient mice exhibited a significant decrease in AHR, as measured by airway resistance (Rn, Figure 2B). Strikingly, serum IgE levels were markedly reduced in Otud6a knockout mice following HDM exposure (Figure 2C). Histopathological analysis via hematoxylin&eosin (H&E) and Periodic Acid‐Schiff (PAS) staining revealed that Otud6a ablation attenuated HDM‐induced inflammatory cell infiltration, epithelial thickening, and goblet cell metaplasia (Figure 2D,E). Furthermore, Otud6a knockout significantly suppressed eosinophil accumulation in bronchoalveolar lavage fluid (BALF, Figure 2F). Otud6a deficiency downregulated T helper 2 (Th2)‐associated cytokines (IL‐5 and IL‐13) in BALF and lung tissues following HDM sensitization and challenge (Figure 2G–L). Quantitative polymerase chain reaction (PCR) confirmed reduced mRNA expression of mucin (*Muc5ac* and *Muc5b*) in HDM‐induced Otud6a knockout mice relative to wild type mice (Figure 2M,N). The alarmins (IL‐25/IL‐33/TSLP) levels in BALF were also decreased in Otud6a knockout mice when compared with wild type asthmatic mice (Figure 2O). Moreover, Otud6a knockout mitigated HDM‐induced epithelial barrier dysfunction, as evidenced by restored expression of tight junction proteins (ZO‐1 and Occludin, Figure 2P; Figure S2A,B). Collectively, these findings demonstrate that OTUD6A deficiency attenuates AHR, airway inflammation, mucin hypersecretion, and epithelial barrier disruption in HDM‐driven acute asthma model.

### 2.3 Otud6a Knockout Alleviates HDM‐Induced AHR and Airway Inflammation in HDM‐Induced Mouse Chronic Asthma Model

**FIGURE 3:**
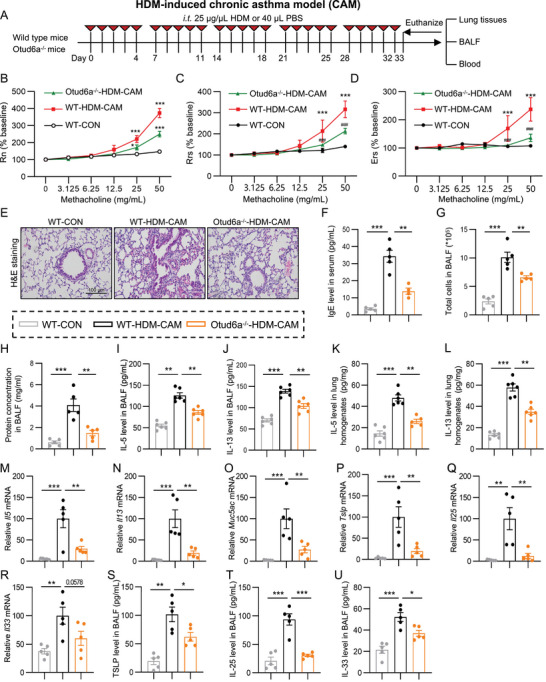
OTUD6A knockout mitigates asthma in HDM‐induced chronic asthma model. A) Schematic diagram depicting the procedure of HDM‐CAM. B‐D) Rn, Ers, and Rrs assessed via methacholine challenge (*n* = 5). E) Representative H&E staining of lung tissue. Scale bars: 100 µm. F) Serum IgE levels measured by ELISA (*n* = 5). G‐H) Total cell counts (G) and protein concentration (H) in BALF (*n* = 5). I‐L) IL‐5 and IL‐13 levels in BALF (I, J) and lung homogenates (K, L) measured by ELISA (*n* = 5). M‐R) RT‐qPCR analysis of *Il5*, *Il13*, *Muc5ac*, *Tslp*, *Il25*, and *Il33* mRNA level in lung tissues (*n* = 5). S‐U) ELISA analysis of IL‐25, IL‐33, and TSLP levels in BALF (*n* = 5). Data are presented as mean ± SEM. *P* values determined by one‐way ANOVA (*p < 0.05, **p < 0.01, ***p < 0.001).

We next investigated the role of Otud6a in chronic asthma model using a prolonged HDM protocol (Figure 3A). Otud6a knockout mice displayed significant reductions in AHR parameters, including Rn (Figure 3B), respiratory rate signal (Rrs, Figure 3C), and expiratory reserve volume (Ers, Figure 3D), compared to HDM‐treated wild‐type mice. The thickness of epithelium and inflammatory cells infiltration in H&E stained lung sections were observed in asthma mice, whereas these changes were remarkably reversed by Otud6a knockout (Figure 3E). Serum IgE levels were also markedly lower in Otud6a knockout cohorts (Figure 3F). Otud6a knockout further led to a significant reduce on the total cell counts and protein concentrations in BALF (Figure 3G,H). Otud6a ablation suppressed Th2 cytokines (IL‐5 and IL‐13) in BALF (Figure 3I,J) and lung homogenates (Figure 3K,L). Quantitive PCR showed that Otud6a deficiency led to a downregulation of Th2 cytokines (*Il5*, *Il13*), mucin (*Muc5ac*), and epithelial‐derived alarms (*Tslp*, *Il25*, and *Il33*) transcripts (Figure 3M–R). HDM‐induced elevation of IL‐25/IL‐33/TSLP in BALF was also decreased due to Otud6a knockout (Figure 3S–U). Collectively, these results suggested that Otud6a knockout attenuates AHR and airway inflammation in chronic asthma model.

### 2.4 OTUD6A Mediates Airway Remodeling Through the PI3K/AKT‐EMT Pathway

**FIGURE 4:**
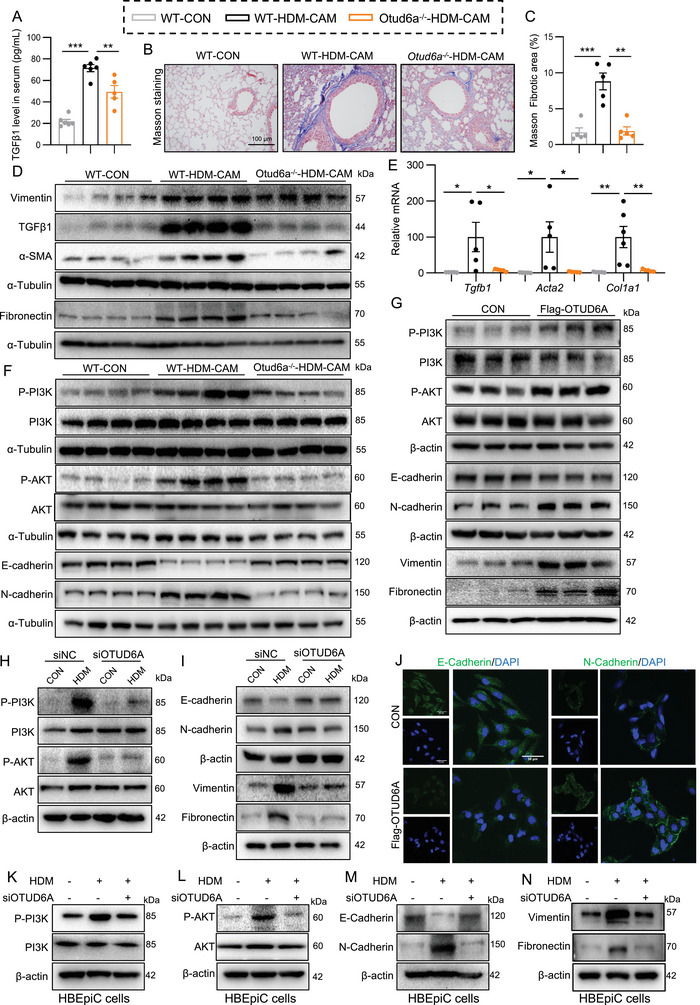
OTUD6A promotes airway remodeling tvia PI3K/AKT‐EMT signaling. **A)** Serum TGFβ1 levels in HDM‐CAM mice (*n* = 5). B‐C) Masson's trichrome staining and quantification of lung sections (*n* = 5). Scale bars: 100 µm. D) Western blot analysis of Vimentin, TGFβ1, α‐SMA, and Fibronectin in lung tissues. E) RT‐qPCR analysis of *Tgfb1*, *Acta2* and *Col1a1* mRNA levels (*n* = 5). F) Western blot analysis of PI3K/AKT and EMT markers in HDM‐CAM lung tissues. G) Western blot analysis of PI3K/AKT, EMT markers, Vimentin, and Fibronectin in BEAS‐2B cells transfected with Flag‐OTUD6A for 24 h. H‐I) Western blot analysis of PI3K/AKT and EMT markers in BEAS‐2B cells transfected with siOTUD6A for 48 h and stimulated with HDM for 6 h. J) Immunofluorescence staining of E‐cadherin and N‐cadherin in BEAS‐2B cells transfected with Flag‐OTUD6A for 24 h. Scale bars: 50 µm. K‐N) HBEpiC cells transfected with siOTUD6A or negative control for 48 h and stimulated with HDM (100 µg/mL) for 6 h. Western blot analysis of PI3K/AKT and EMT markers. Data are presented as mean ± SEM. *P* values determined by one‐way ANOVA (*p < 0.05, **p < 0.01, ***p < 0.001).

Airway remodeling is a key pathological feature of asthma, so we investigated the effect of OTUD6A on airway remodeling. Notably, Otud6a knockout attenuated airway remodeling, as demonstrated by the reduced serum transforming growth factor β1 (TGFβ1) levels (Figure 4A), decreased collagen deposition (Masson's trichrome, Figure 4B,C), and downregulated the expression of fibrotic protein (Vimentin, TGFβ1, α‐smooth muscle actin (α‐SMA), and Fibronectin, Figure 4D; Figure S3A–D) and mRNA (*Tgfb*, *Acta2* and *Col1a1*, Figure 4E). These data conclusively establish that Otud6a deficiency protects against HDM‐induced airway remodeling.

Given that EMT in AECs is a critical pathological mechanism underlying airway remodeling [20, 21], and considering that key mesenchymal markers (Vimentin, α‐SMA, Fibronectin) were already suppressed by Otud6a knockout (Figure 4D), we investigated the effects of OTUD6A on EMT progression and the activation of its key regulatory effector, the phosphatidylinositol 3 kinase/protein kinase B (PI3K/AKT) pathway [22]. In chronic asthmatic murine lung tissues, we observed elevated phosphorylation levels of PI3K and AKT, concomitant with reduced expression of the epithelial marker E‐cadherin and upregulation of the mesenchymal marker N‐cadherin (Figure 4F; Figure S3E–H). Strikingly, genetic ablation of OTUD6A markedly suppressed PI3K/AKT pathway activation and attenuated EMT progression (Figure 4F; Figure S3E–H). In vitro studies further demonstrated that overexpression of OTUD6A enhanced PI3K/AKT phosphorylation and promoted EMT (Figure 4G; Figure S3I–N), whereas it's silencing effectively blocked HDM‐induced PI3K/AKT activation and EMT (Figure 4H,I; Figure S3O–T) in BEAS‐2B cells. Immunofluorescence analysis corroborated these findings, revealing diminished E‐Cadherin fluorescence and intensified N‐Cadherin signals upon OTUD6A overexpression (Figure 4J). To substantiate the pathophysiological relevance of these findings, we extended our investigation to human primary airway epithelial cells (HBEpiC). Consistent with the observations in BEAS‐2B cells, OTUD6A overexpression similarly enhanced the activation of PI3K/AKT pathway and EMT (Figure S4A–H). Conversely, OTUD6A silencing potently inhibited HDM‐induced activation of these processes in HBEpiCs (Figure 4K–N; Figure S4I–N). Taken together, these results indicated that OTUD6A mediates airway remodeling through PI3K/AKT‐EMT pathway.

### 2.5 OTUD6A Mediates hResistin/mRELMα Expression

**FIGURE 5:**
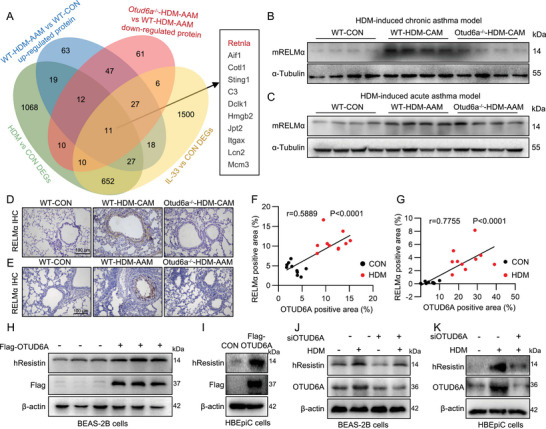
hResistin/mRELMα is a potential substrate of OTUD6A. A) Multi‐omics identification of OTUD6A substrates. B‐C) Western blot of mRELMα expression in HDM‐CAM (B) and HDM‐AAM (C) lung tissues. D‐E) IHC staining of mRELMα in HDM‐CAM (D) and HDM‐AAM (E) lung tissues. Scale bars: 100 µm. F‐G) Correlation analysis of mRELMα and OTUD6A IHC staining in HDM‐CAM (F) and HDM‐AAM (G). Two random fields per mouse. H) Western blot analysis of hResistin in BEAS‐2B cells transfected with Flag‐OTUD6A and control vector for 24 h. I) Western blot analysis of hResistin in HBEpiC cells transfected with Flag‐OTUD6A and control vector for 24 h. J) Western blot analysis of hResistin in BEAS‐2B cells transfected with siOTUD6A for 24 h with or without HDM (100 µg/mL) treatment for 6 h. K) Western blot analysis of hResistin in HBEpiC cells transfected with siOTUD6A for 24 h with or without HDM (100 µg/mL) treatment for 6 h. Data are presented as mean ± SEM. *P* values determined by two‐tailed unpaired t‐test or one‐way ANOVA (*p < 0.05, **p < 0.01, ***p < 0.001).

To delineate the molecular mechanisms underlying OTUD6A‐mediated asthma pathogenesis, we performed quantitative proteomic analysis of lung tissues from HDM‐induced acute asthma model. Comparative profiling revealed 224 upregulated proteins in HDM‐challenged wild‐type mice versus unchallenged controls. Strikingly, Otud6a knockout suppressed the expression of 184 of these HDM‐induced proteins. Intersectional analysis with our prior transcriptomic datasets from murine asthma (HDM‐induced) and airway inflammation (IL‐33‐induced) models identified 11 overlapping protein‐coding genes (Figure 5A). Among these, *Retnla* exhibited the most pronounced differential expression (Figure S5A), implicating it may be a key downstream effector of OTUD6A‐mediated regulation in asthma.

Consistent with proteomic findings, we validated the expression of mRELMα (encoded by *Retnla*) across chronic and acute murine asthma models. Immunoblotting demonstrated significant upregulation of mRELMα protein in HDM‐challenged lungs versus controls (Figure 5B,C; Figure S5B,C), which was attenuated by Otud6a knockout. Immunohistochemistry staining confirmed OTUD6A‐dependent mRELMα expression and mRELMα positive signals predominantly localized to airway epithelial area (Figure 5D,E). Quantification revealed strong correlation between OTUD6A and mRELMα positivity (HDM‐CAM, Pearson's r = 0.5889, *p* < 0.0001, Figure 5F; HDM‐AAM, Pearson's r = 0.7755, *p* < 0.0001, Figure 5G), suggesting co‐regulation in disease‐relevant niches. Notably, human lack an ortholog of murine RELMα (*Retnla*), necessitating investigation of its evolutionarily conserved paralog Resistin [23, 24]. OTUD6A overexpression significantly upregulated hResistin protein levels both in BEAS‐2B (Figure 5H; Figure S5D) and HBEpiC (Figure 5I; Figure S5E) cells. Conversely, OTUD6A siRNA reduced HDM‐induced hResistin expression both in BEAS‐2B (Figure 5J; Figure S5F) and HEBpiC (Figure 5K; Figure S5G) cells, mirroring murine mRELMα regulation patterns. Taken together, these results indicated that OTUD6A regulated hResistin/mRELMα expression in vivo and in vitro.

### 2.6 hResistin/PI3K Mediates OTUD6A‐Induced EMT Process and the Expression of Epithelial‐Derived Alarmins

**FIGURE 6:**
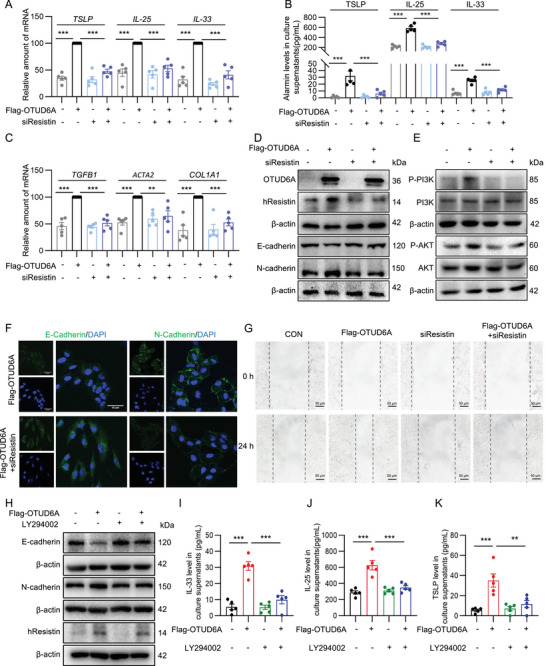
hResistin/PI3K mediates OTUD6A‐induced EMT process and the expression of epithelial‐derived alarmins. A‐G) BEAS‐2B cells transfected with siResistin for 48 h and then transfected with Flag‐OTUD6A for 24 h. A) RT‐qPCR analysis of *TSLP*, *IL‐25*, *IL‐33* mRNA level in BEAS‐2B cells (*n* = 5). B) ELISA analysis of TSLP, IL‐25, and IL‐33 levels in cell supernatant (*n* = 5). C) RT‐qPCR analysis of *TGFB1*, *ACTA2*, *COL1A1* mRNA level in BEAS‐2B cells (*n* = 5). D‐E) Western blot analysis of OTUD6A, hResistin, EMT, and PI3K/AKT markers. F) Immunofluorescence staining of E‐cadherin and N‐cadherin. Scale bars: 50 µm. G) Cell wound healing assay. Scale bars: 50 µm. H‐K) BEAS‐2B cells were pretreated with LY294002 (30 µm) for 30 min and transfected with Flag‐OTUD6A for 24 h. H) Western blot analysis of hResistin and EMT markers. I‐K) ELISA analysis of IL‐33, IL‐25, and TSLP levels in cell supernatant (*n* = 5). Data are presented as mean ± SEM. *P* values determined by one‐way ANOVA (*p < 0.05, **p < 0.01, ***p < 0.001).

To further delineate the downstream mediators of OTUD6A‐induced epithelial injury in vitro, we investigated the functional role of hResistin. Genetic silencing of hResistin potently attenuated the OTUD6A‐driven pathological cascade, including: suppressed gene expression and secretion of epithelial‐derived alarms (TSLP, IL‐25 and IL‐33, Figure 6A,B), downregulation gene of fibrogenic markers (*TGFB1*, *COL1A1* and *ACTA2*, Figure 6C), reversal of EMT progression (Figure 6D; Figure S6A,B), inhibition of PI3K/AKT phosphorylation (Figure 6E; Figure S6C,D), and impaired epithelial cell migratory capacity in wound healing assays (Figure 6G). Immunofluorescence analysis also revealed that hResistin silence markedly enhanced E‐Cadherin fluorescence intensity while concomitantly reducing N‐Cadherin signal intensity compared with OTUD6A‐overexpressing cells (Figure 6F). Additionally, we detected the mediated role of PI3K in this process through using its inhibitor LY294002. The results showed that PI3K inhibition significantly reduced OTUD6A overexpression‐induced EMT (Figure 6H; Figure S6E,F) and alarmins secretion (Figure 6I–K), but had no effect on Resistin expression (Figure 6H; lower two panels, Figure S6G), indicating PI3K was the downstream of Resistin. These collective findings established that hResistin/PI3K as the critical downstream executor mediating OTUD6A‐dependent epithelial damage.

### 2.7 OTUD6A Deubiquitinates hResistin in the Endoplasmic Reticulum to Divert It from Proteasomal Degradation to Secretion

**FIGURE 7:**
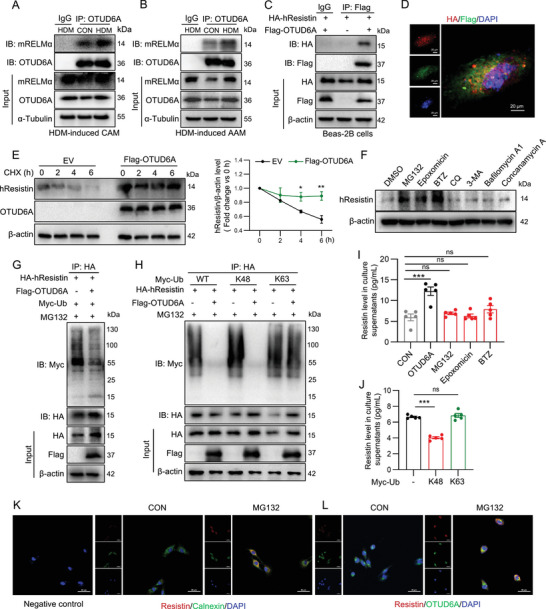
OTUD6A deubiquitinates hResistin in the endoplasmic reticulum to divert it from proteasomal degradation to secretion. A‐B) Co‐immunoprecipitation of endogenous OTUD6A and mRELMα in HDM‐CAM (A) and HDM‐AAM (B) lung tissues. C) Co‐immunoprecipitation of exogenous OTUD6A and hResistin in BEAS‐2B cells expressed Flag‐OTUD6A and HA‐hResistin. D) Immunofluorescence of hResistin and OTUD6A in BEAS‐2B cells transfected with Flag‐OTUD6A and HA‐hResistin. Scale bars: 20 µm. **E)** CHX chase assay for hResistin stability (*n* = 3). F) BEAS‐2B cells were stimulated with DMSO, MG132 (10 µm), Epoxomicin (2.5 µm), BTZ (5 µm), CQ (25 µm), 3‐MA (10 mm), Bafilomycin A1 (1 µm), or Concanamycin A (0.5 µm) for 6 h. Western blot analysis of hResistin in BEAS‐2B cells. **G)** HA‐hResistin, Flag‐OTUD6A, and Myc‐Ub were transfected into BEAS‐2B cells and then subjected to 10 µm MG132 for 6 h. Ubiquitinated hResistin was detected. H) HA‐hResistin, Flag‐OTUD6A, Myc‐WT Ub, Myc‐K48 Ub, and Myc‐K63 Ub were transfected into BEAS‐2B cells and then subjected to 10 µm MG132 for 6 h. Ubiquitinated hResistin was detected. I) BEAS‐2B cells were stimulated with DMSO, MG132 (10 µm), Epoxomicin (2.5 µm), or BTZ (5 µm) for 6 h or transfected with Flag‐OTUD6A for 24 h, ELISA analysis of hResistin levels in cell supernatant (*n* = 5). J) BEAS‐2B cells transfected with Myc‐K48 Ub or Myc‐K63 Ub for 24 h, ELISA analysis of hResistin levels in cell supernatant (*n* = 5). K) Immunofluorescence of Resistin and Calnexin in BEAS‐2B cells treated with MG132 (10 µm) or not for 6 h. Scale bars: 50 µm. L) Immunofluorescence of Resistin and OTUD6A in BEAS‐2B cells treated with MG132 (10 µm) or not for 6 h. Scale bars: 50 µm. Data are presented as mean ± SEM. *P* values determined by one‐way ANOVA (*p < 0.05, **p < 0.01, ***p < 0.001).

As a deubiquitinating enzyme, OTUD6A modulates cellular processes through direct PTM of substrate proteins [15, 25]. To elucidate the mechanistic basis of OTUD6A function in asthma pathogenesis, we systematically examined its potential physical interaction with hResistin/mRELMα. Strikingly, co‐immunoprecipitation analyses of lung tissue lysates revealed robust complex formation between OTUD6A and mRELMα, with this interaction being consistently observed across two distinct experimental asthma models (Figure 7A,B). This interaction was conserved in human AECs, as evidenced by OTUD6A‐hResistin binding in BEAS‐2B cells (Figure 7C). Immunofluorescence analysis confirmed co‐localization of OTUD6A and hResistin in BEAS‐2B cells (Figure 7D), suggesting hResistin/mRELMα as a potential substrate protein of OTUD6A.

To characterize the functional consequences of this interaction, we assessed hResisitin protein stability. Cycloheximide (CHX) chase experiments revealed that OTUD6A overexpression significantly decreased the degradation rate of endogenous hResistin (Figure 7E), while quantitative RT‐ qPCR showed no corresponding changes in *RETN* (coding hResistin) mRNA in BEAS‐2B cells (Figure S7A). Consistently, Otud6a knockout did not alter *Retnla* transcription in asthmatic murine lungs (Figure S7B,C).

To define the degradation pathway responsible for hResistin turnover and its regulation by OTUD6A, we employed pharmacological inhibitors. In control cells, proteasomal inhibitors (MG132, Epoxomicin, BTZ), but not lysosomal/autophagy inhibitors (CQ, 3‐MA, Bafilomycin A1, Concanamycin A), robustly increased intracellular hResistin levels (Figure 7F), establishing the proteasomal pathway as the primary route for its degradation. It should be noted that although CQ and 3‐MA were also able to increase the protein level of hResistin, the inconsistency of data ability indicates that the autophagy/lysosomal pathway may affect the stability of hResistin but is not the main pathway. Ubiquitination assays revealed that OTUD6A overexpression substantially reduced hResistin polyubiquitination in BEAS‐2B cells (Figure 7G). Using ubiquitin mutants retaining only K48 or K63 linkage sites, we found OTUD6A specifically cleaved K48‐linked polyubiquitin chains (Figure 7H), suggesting OTUD6A regulates hResistin deubiquitination in a K48‐linked manner.

As hResistin is a secretory protein, we further investigated the effects of OTUD6A and proteasome inhibitors on hResistin secretion. Intriguingly, while OTUD6A overexpression markedly enhanced the secretion of hResistin (Figure 7I), pharmacological blockade of the proteasome with MG132, Epoxomicin, and BTZ did not. This indicated that OTUD6A promotes secretion not by generically inhibiting degradation, but by actively redirecting hResistin fate. Overexpression of a ubiquitin mutant retaining only K48 linkages (K48 only Ub) significantly reduced the secretion of hResistin, whereas K63‐only Ub has no such effect (Figure 7J), indicating OTUD6A promotes hResistin secretion through its catalytic removal of K48‐linked polyubiquitin chains. Subcellular localization analysis revealed that upon proteasomal inhibition (MG132), hResistin strongly accumulated and co‐localized with the endoplasmic reticulum (ER) marker Calnexin (Figure 7K). Importantly, under the identical treatment condition, OTUD6A also showed marked co‐localization with the accumulated pool of hResistin (Figure 7L). These data establish that OTUD6A deubiquitinates and stabilizes hResistin within the ER, likely rescuing it from a degradative fate such as ER‐associated degradation (ERAD) and facilitating its subsequent entry into the secretory pathway.

### 2.8 OTUD6A Requires Its Intact OTU Domain and Conserved C152 Residue to Deubiquitinate hResistin at K2 and K19

**FIGURE 8:**
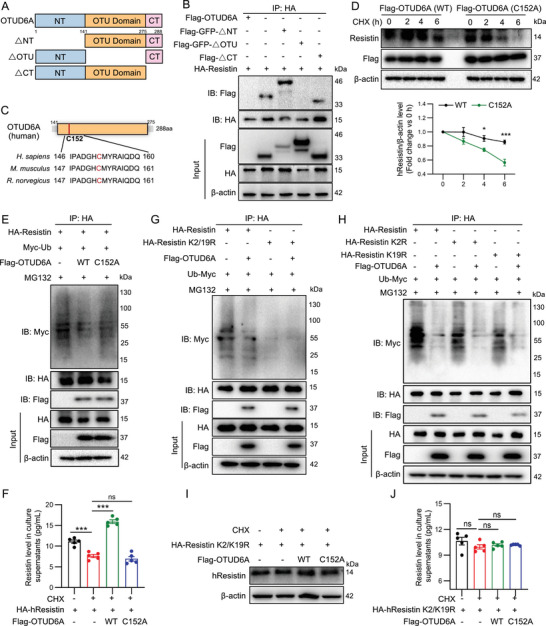
OTUD6A undergoes deubiquitination at K2 and K19 of hResistin through its OTU domain and C152 residue. A) Schematic diagram of OTUD6A and its truncated mutants. B) Flag‐OTUD6A or its truncated mutants and HA‐hResistin transfected into BEAS‐2B cells. Mapping of OTUD6A‐hResistin interaction domains. C) Conserved catalytic cysteine (C152A) in OTUD6A orthologs. D) BEAS‐2B cells transfected with Flag‐OTUD6A (WT or C152A), following CHX (100 µg/mL) treatment for the indicated times. CHX chase assay for hResistin stability. E) HA‐hResistin and Myc‐Ub were transfected into BEAS‐2B cells together with Flag‐OTUD6A (WT or C152A) and then subjected to 10 µm MG132 for 6 h. Ubiquitinated hResistin was detected. F) HA‐hResistin was transfected into BEAS‐2B cells together with Flag‐OTUD6A (WT or C152A) and then subjected to CHX (100 µg/mL) for 6 h. ELISA analysis of hResistin levels in cell supernatant (*n* = 5). G‐H) HA‐hResistin (WT, K2R, K19R or K2/K19R), Flag‐OTUD6A, and Myc‐Ub were transfected into BEAS‐2B cells and then subjected to 10 µm MG132 for 6 h. Ubiquitinated hResistin was detected. I‐J) HA‐hResistin (K2/K19R) and Flag‐OTUD6A (WT or C152A) were transfected into BEAS‐2B cells for 24 h, and then subjected to CHX (100 µg/mL) for 6 h. I) Western blot analysis of hResistin in BEAS‐2B cells. J) ELISA analysis of hResistin levels in cell supernatant (*n* = 5). Data are presented as mean ± SEM. *P* values determined by one‐way ANOVA (*p < 0.05, **p < 0.01, ***p < 0.001).

To determine the key structural domain of OTUD6A regulating hResistin deubiquitination, truncated mutants for OTUD6A were constructed (Figure 8A). While full‐length OTUD6A and constructs lacking either the N‐terminal (ΔNT) or C‐terminal (ΔCT) domains retained binding capacity, the OTU domain deletion mutant (ΔOTU) failed to interact (Figure 8B). Sequence alignment revealed evolutionary conservation of the catalytic cysteine residue (C152) across species including *Homo sapiens*, *Mus musculus*, and *Rattus norvegicus* (Figure 8C) [13]. The C152A mutation abrogated OTUD6A's ability to stabilize hResistin (Figure 8D), significantly impaired its deubiquitinating activity (Figure 8E), and the secretion of hResistin (Figure 8F). This establishes C152 as the critical catalytic residue for OTUD6A's enzymatic function.

The hResistin protein sequence contains only two lysine residues (K2 and K19). To investigate the ubiquitination site mediated by OTUD6A, we constructed plasmids carrying single and double mutations. K2/K19R, K2R, and K19R mutation did not affect the binding between OTUD6A and hResistin (Figure 8G,H). The ubiquitination modification of hResistin disappeared when both lysine residues were mutated (Figure 8G, lane 3), whereas the two single mutations could only partially reverse the ubiquitination (Figure 8H, line 3, 5). However, OTUD6A overexpression could completely reverse the remaining ubiquitination of K2R and K19R of hResistin (Figure 8H, line 4, 6). The double mutation was inherently more stable than wild‐type hResistin, as CHX treatment after 6 h had no effect on the stability of the double mutant protein (Figure 8I, line 2). Importantly, neither wild‐type OTUD6A nor the C152A catalytic mutant could further alter the intracellular protein level of hResistin with double mutations. Parallel measurement of secreted hResistin confirmed that OTUD6A lost its ability to promote the secretion of hResistin with double mutations (Figure 8J). Collectively, these results establish that K2 and K19 are functionally necessary and sufficient for OTUD6A‐mediated regulation of hResistin.

### 2.9 Lung Tissues OTUD6A Specific Knockout Attenuated HDM‐Induced Chronic Asthma in Mice

**FIGURE 9:**
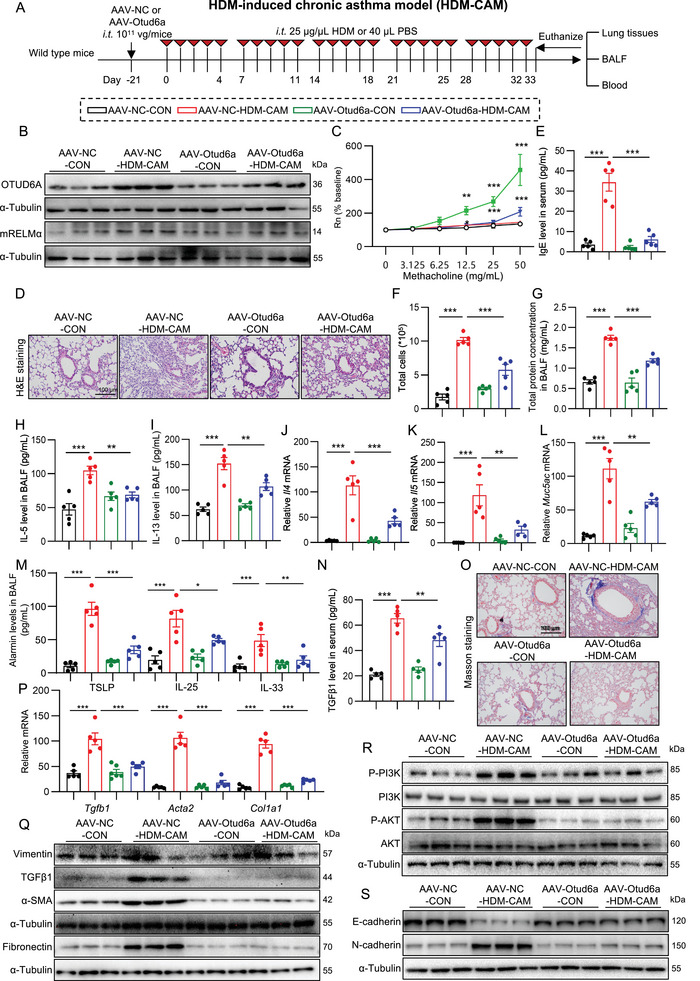
Lung‐specific OTUD6A knockdown attenuates HDM‐induced asthma. A) Schematic diagram depicting the procedure of HDM‐induced chronic asthma model. B) Western blot analysis of OTUD6A and mRELMα in lung tissues. C) AHR assessed via acetylcholine challenge (*n* = 5). D) H&E staining of lung tissues. Scale bars: 100 µm. E) Serum IgE levels measured by ELISA (*n* = 5). F‐G) Total cell counts (F) and protein concentration (G) in BALF (*n* = 5). H‐I) IL‐5 (H) and IL‐13 (I) levels in BALF measured by ELISA (*n* = 5). J‐L) RT‐qPCR analysis of *Il4, Il5*, and *Muc5ac* mRNA levels in lung tissues (*n* = 5). M) ELISA analysis of IL‐25, IL‐33, and TSLP levels in BALF (n = 5). N) Serum TGF‐β1 levels measured by ELISA (*n* = 5). O) Masson's trichrome staining of lung sections. Scale bars: 100 µm. P) RT‐qPCR analysis of *Tgfb1*, *Acta2*, and *Col1a1* mRNA levels in lung tissues (*n* = 5). Q) Western blot analysis of Vimentin, TGFβ1, α‐SMA, and Fibronectin in the lung tissues. R) Western blot analysis of PI3K/AKT in the lung tissues. S) Western blot analysis of EMT markers in the lung tissues. Data are presented as mean ± SEM. *P* values determined by one‐way ANOVA (*p < 0.05, **p < 0.01, ***p < 0.001).

To elucidate whether OTUD6A in lung tissue mediates HDM‐induced asthma, we employed Adeno‐associated virus serotype 6 (AAV6) to specifically knockdown OTUD6A expression in murine asthma pulmonary tissues (Figure 9A). Western blotting bands showed that AAV‐Otud6a treatment significantly reduced the basal level of OTUD6A, as well as in asthmatic mice (Figure 9B; Figure S8A). The elevation of mRELMα in asthmatic mice was also reduced by Otud6a knockdown (Figure 9B; Figure S8B). Lung‐specific Otud6a knockdown significantly attenuated asthma‐related pulmonary function parameters including Rn, Rrs, and Ers in murine models (Figure 9C; Figure S8C,D). Histopathological examination via H&E staining revealed that Otud6a knockdown markedly ameliorated hallmark asthmatic pathologies including airway epithelial thickening and inflammatory cell infiltration (Figure 9D). Serum IgE levels (Figure 9E), total inflammatory cell counts in BALF (Figure 9F), and protein concentrations in BALF (Figure 9G) were concomitantly reduced following Otud6a depletion. The intervention substantially suppressed Th2‐associated responses, demonstrated by decreased protein (IL‐5 and IL‐13) and transcriptional (*Il4* and *Il5*) expression levels in both BALF and lung tissues (Figure 9H–K; Figure S8E,F), reduced *Muc5ac* gene expression (Figure 9L), as well as the gene level (Figure S8G) and the protein expression in BALF (Figure 9M) of epithelial‐derived alarmins (IL‐25, IL‐33, TSLP). Furthermore, lung‐specific Otud6a knockdown significantly mitigated HDM‐induced airway remodeling, as evidenced by reduced serum TGFβ1 levels (Figure 9N), diminished collagen deposition (Figure 9O, Figure S8H), downregulation of fibrotic markers at both mRNA (*Tgfb1*, *Acta2* and *Col1a1*; Figure 9P) and protein (Vimentin, α‐SMA, TGF‐β1, Fibronectin; Figure 9Q; Figure S8I–L) levels, inhibition of PI3K/AKT signaling pathway activation (Figure 9R; Figure S8M,N), and blockade of EMT progression (Figure 9S; Figure S8O,P). Collectively, these findings demonstrate that pulmonary‐specific OTUD6A knockdown alleviates asthma pathogenesis through comprehensive suppression of HDM‐induced AHR, airway inflammation, and airway remodeling.

## 3 Discussion

Our study establishes OTUD6A as a previously unrecognized, pathogenic driver in asthma. We demonstrate that OTUD6A expression is specifically upregulated in AECs of asthmatic patients and murine models. Critically, genetic ablation of Otud6a not only attenuated AHR, airway inflammation, and mucus overproduction but also mitigated airway remodeling in chronic settings, establishing its central role across asthma endotypes. To unravel the underlying mechanism, we employed multi‐omics screening and identified hResistin (and its murine homolog mRELMα) as a key substrate of OTUD6A in AECs. At the molecular level, OTUD6A directly and specifically removes K48‐linked polyubiquitin chains from hResistin at lysine K2 and K19 via its catalytic site C152. This deubiquitination consequently shields hResistin from proteasomal degradation, leading to its aberrant accumulation and secretion. Collectively, our work delineates a complete pathogenic axis‐from OTUD6A overexpression to hResistin stabilization‐in asthma pathogenesis, and nominates OTUD6A as a compelling therapeutic target for intervention.

AECs dysfunction has been definitively established as a cornerstone of asthma pathogenesis, operating through two principal mechanisms [3]. First, AECs secrete epithelial‐derived alarmins (e.g., IL‐33, TSLP) that amplify both innate and adaptive type 2 immune responses [26, 27]. Second, pathological basal cell differentiation‐manifesting as goblet cell hyperplasia and EMT‐directly impairs mucociliary clearance and promotes airway remodeling [28]. Supporting this paradigm, Werder and colleagues identified that activation of the purinergic receptor P2Y(13) promotes nuclear translocation and subsequent release of IL‐33 and high mobility group box‐1 (HMGB1), exacerbating experimental asthma [29]. Furthermore, integrin beta 4 (ITGB4) deficiency in AECs has been shown to potentiate airway remodeling via activation of epithelial‐mesenchymal trophic units, mediated through the Src homology domain 2‐containing protein tyrosine phosphatase 2/c‐Jun N‐terminal kinase/Jun N‐terminal kinase‐dependent transcription factor/fibroblast growth factor 2 (SHP2/JNK/c‐Jun/FGF2) signaling axis [30]. Our findings significantly extend this conceptual framework by demonstrating that OTUD6A in AECs critically regulates hResistin/mRELMα‐mediated alarmin production and contributes to airway remodeling processes.

DUBs exhibit complex, context‐dependent roles in asthma. While certain USPs (e.g., USP7, USP38, USP22) exacerbate type 2 inflammation by stabilizing key signaling molecules like STAT3 [11] or JunB [12] to enhance Th2 cell differentiation and cytokine production, or bromodomain‐containing protein 4 (BRD4) to drive airway smooth muscle proliferation and remodeling [31], others like A20/TNFAIP3 act as suppressors [32, 33]. Although OTUD6A is classically characterized as an oncogene promoting tumor progression and chemoresistance [25], recent studies implicate it in innate immunity and inflammatory bowel disease via substrate stabilization [15, 18]. Our discovery of OTUD6A's pro‐asthmatic function expands this regulatory landscape Notably, its reported functions can appear divergent‐for instance, it can suppress LPS‐induced interferon in innate immune contexts [18], yet promote inflammation in others [15, 16]. This underscores that OTUD6A, like many DUBs, is a context‐dependent regulator whose net effect is determined by cell type, stimulus, and specific substrates engaged. However, its role in asthma remained unexplored. We demonstrate that OTUD6A is highly expressed in asthmatic AECs, and its genetic deletion attenuates HDM‐induced AHR, airway inflammation, and airway remodeling. More importantly, OTUD6A directly regulates epithelial‐derived alarms and EMT in human AECs, establishing its centrality in asthma pathogenesis.

The RELMs family exhibits species‐specific heterogeneity: rodents express four members (RELMα, RELMβ, Resistin, and RELMγ) versus two in humans (Resistin, RELMβ) [34]. Notably, hResistin shares functional homology with mRELMα rather than mResistin in inflammatory modulation [24, 25, 35, 36]. Both proteins act as Th2 amplifiers in allergic asthma, potentially via damage‐associated molecular patterns‐receptor for advanced glycation end‐products (DAMP‐RAGE) or toll‐like receptor 4 (TLR4) signaling [34, 37, 38]. Our genomic analyses revealed that *Retnla* (coding mRELMα) as the most upregulated gene in asthmatic mice, with corroborating protein‐level increases in lung tissues. Importantly, OTUD6A‐dependent hResistin stabilization drove epithelial alarmin production, while hResistin silence abrogated OTUD6A‐induced EMT in Beas‐2B cells‐despite having no effect under basal conditions. These data position hResistin as the critical downstream effector of OTUD6A in Th2 inflammation and airway remodeling, consistent with reports that mRELMα (found in inflammatory zone 1, FIZZ1) promotes pathological airway restructuring [39, 40]. However, in our cellular model, pharmacological inhibition of RAGE or TLR4 failed to attenuate OTUD6A‐induced EMT and alarmins secretion (Figure S9). These results indicate that the receptor mechanism mediating OTUD6A/hResistin signaling in this specific context is distinct from the canonical pathways, and remains to be identified.

While OTUD6A has been implicated as a regulator in various contexts [15, 25], our study revealed its previously unknown role in asthma by identifying hResistin/mRELMα as its key substrate in airway epithelium. Notably, the post‐translational regulation of this pivotal asthma‐related cytokine, particularly its turnover via the ubiquitin‐proteasome system (UPS), has been largely unexplored. We have delineated a complete UPS‐based regulatory axis in AECs: OTUD6A, via its catalytic residue Cys152, directly cleaves K48‐linked polyubiquitin chains on hResistin at lysines K2 and K19. As a secretory protein, hResistin is likely subject to quality control pathways such as ERAD, which typically retro‐translocates proteins from the ER for proteasomal destruction [41, 42]. Our data‐including K48‐linkage specificity, ER localization upon proteasomal inhibition, and the functional separation of degradation from secretion‐are consistent with a model wherein OTUD6A‐mediated deubiquitination antagonizes this ERAD‐like pathway. This action thereby rescuing hResistin from proteasomal degradation and enabling its pathological accumulation and secretion. This mechanistic insight is consistent with the established role of Cys152 in OTUD6A's enzymatic activity [13] and supports a model in which ERAD‐mediated proteasomal regulation serves as a major clearance route for hResistin in AECs. The precise binding interface and mechanistic details underlying OTUD6A‐hResistin interaction remain an open question. Future structural studies will be invaluable in elucidating the molecular architecture of this enzyme‐substrate complex and its deubiquitination kinetics, information that could guide the rational design of inhibitors targeting this asthma‐specific DUB‐substrate axis.

Allergic asthma represents the predominant endotype of asthma, with diverse triggers including HDM, albumin, fungal allergens (e.g., Alternaria), and cytokine mediators such as IL‐33 [43]. Our data reveal that OTUD6A expression is consistently upregulated across multiple experimental models of allergic airway inflammation‐not only in HDM‐induced asthma but also in OVA‐driven allergic responses and Alternaria/IL‐33‐triggered inflammation. To interrogate the broad‐spectrum regulatory role of OTUD6A in allergic asthma pathogenesis, we employed an OVA sensitization‐challenge model in Otud6a knockout mice. Strikingly, genetic ablation of Otud6a significantly attenuated hallmark features of allergic asthma, including AHR, inflammatory cell infiltration, and mucin hypersecretion (Figure S10). These findings position OTUD6A as a central mediator of allergic inflammation across divergent triggers, suggesting its mechanistic role extends beyond specific allergen recognition pathways.

In summary, this study delineates a previously unrecognized post‐translational regulatory axis that drives allergic asthma pathogenesis: the OTUD6‐hResistin axis in airway epithelial cells (AECs). We demonstrated that OTUD6A stabilizes hResistin in AECs by specifically removing its K48‐linked ubiquitin chains, thereby preventing its degradation, likely via the ERAD pathway. The consequent accumulation of hResistin driving two pivotal disease‐promoting processes: the initiation of epithelial‐derived alarmin responses and the progression of EMT. Given this central mechanistic role, pharmacological inhibition of OTUD6A emerges not merely as a novel strategy, but as a rationally targeted therapeutic approach aimed at intercepting a proximal driver of the disease. While this study employed female mice as a standard model for allergic asthma, we note that future investigations including both sexes and including contemporaneous immunofluorescence controls with further strengthen the generalizability and precision of the findings. This work therefore provides a compelling mechanistic rationale and a specific molecular target for future preclinical and therapeutic development in asthma.

## 4 Material and Methods

### 4.1 Cell Culture

The human bronchial epithelial cell line BEAS‐2B (RRID: CVCL_0168, Strain: N/A, Clone number: N/A, Cat# STCC10202G) were purchased from Servicebio (Wuhan, China). The cells were cultured in DMEM (Gibco, Eggenstein, Germany) with 10% fetal bovine serum (FBS, TOCYTO, Colorado, USA) and 1% penicillin/streptomycin in a humidified atmosphere at 37°C with 5% CO_2_.

The human primary bronchial epithelial cells (HBEpiC; RRID: N/A, Strain: N/A, Clone number: N/A, Cat# Delf‐10635) were purchased from Hefei Wanwu Biotechnology Co., LTD (Hefei, China). The cells were cultured in a special medium (Cat# Delf‐25428, Hefei Wanwu Biotechnology Co., LTD, Hefei, China) with 10% FBS (TOCYTO, Colorado, USA) and 1% penicillin/streptomycin in a humidified atmosphere at 37°C with 5% CO_2_.

### 4.2 Reagents

Dermatophagoides pteronyssinus (HDM, Cat# XPB91D3A25) and Alternaria alternata extracts (Alt, Cat# XPMID3A25) were purchased from GREER (Lenoir, NC, USA). Albumin from chicken egg white (OVA, Cat# A5503) was bought from Sigma–Aldrich (St Louis, MO, USA). Recombinant murine protein IL‐33 (rmIL‐33, Cat# CG73) was purchased from novoprotein (Shanghai, China).

Mouse IL‐5 Enzyme Linked Immunosorbent Assay (ELISA) Kit (Cat# 88‐7054‐88), Mouse IL‐13 ELISA Kit (Cat# 88‐7137‐88), and Mouse IgE ELISA Kit (Cat# 88‐50460‐88) were obtained from Thermo Fisher (CA, USA). Human IL‐25 (Cat# E‐EL‐H1648)/IL‐33 (E‐EL‐H2402)/TSLP (Cat# E‐EL‐H1598)/Resistin (Cat# E‐EL‐H1213) ELISA kits, and mouse IL‐25 (Cat# E‐EL‐M0187)/IL‐33 (Cat# E‐EL‐M2642)/TSLP (Cat# E‐EL‐M0642) ELISA kits were purchased from Elabscience (Wuhan, China). Pan‐species TGF‐β1 ELISA kit (Cat# EH0012) was bought from HUABIO (Hangzhou, China). H&E Stain Kit (Cat# G1120), PAS Stain Kit (Cat# G1285), and Masson's Trichrome Stain Kit (Cat# G1340) were purchased from Solarbio Science & Technology (Beijing, China).

Antibodies for β‐actin (Cat# 66009‐1‐Ig), α‐Tubulin (Cat# 14555‐1‐AP), OTUD6A (Cat# 24486‐1‐AP), HA‐Tag (Cat# 51064‐2‐AP), Flag‐Tag (Cat# 20543‐1‐AP), Myc‐Tag (Cat# 60003‐2‐Ig) and α‐SMA (Cat# 14395‐1‐AP) were obtained from Proteinthch (Wuhan, China); Antibodies for p‐PI3K (Cat# 17366), PI3K p85 (Cat# 4292), p‐AKT (Cat# 4060), and AKT (Cat# 9272) were bought from Cell Signaling Technology (Beverly, MA, USA); ZO‐1 (Cat# ab307799) and Occludin (Cat# ab216327) antibodies were acquired from Abcam (Cambridge, MA, USA); TGF‐β1 (Cat# WL02998) and Vimentin (Cat# WL01960) antibodies were sourced from Wanleibio (Shenyang, China); E‐Cadherin (Cat# AF0131), N‐Cadherin (Cat# AF5239), and Calnexin (Cat# AF5362) antibody were purchased from Affinity (Nanjing, China); Antibody for hResistin (Cat# ME4290212) was acquired from Abmart (Shanghai, China). Antibody for mRELMα (Cat# MAB1523) was obtained from R&D Systems (Minneapolis, MN, USA). HRP‐linked second Antibody (Cat# A0208, Cat# A0350), Protein A + G Agarose (Cat# P2055), and Fibronentin (Cat# AF6912) were purchased from Beyotime (Shanghai, China); Cycloheximide (CHX, Cat# HY‐12320), MG132 (Cat# HY‐13259), Bafilomycin A1 (Cat# HY‐100558), Concanamycin A (Cat# HY‐N1724), Epoxomicin (Cat# HY‐13821), Bortezomib (BTZ, Cat# HY‐10227), LY294002 (Cat# HY‐10108), FPS‐ZM1 (Cat# HY‐19370), and TAK‐242 (Cat# HY‐11109) were sourced from MCE (Shanghai, China). 3‐Methyladenine (3‐MA, Cat# T1879) were acquired from TargetMol (shanghai, China). Chloroquine (CQ, Cat# 085M4098V) were obtained from Sigma–Aldrich (St Louis, MO, USA).

### 4.3 Plasmids Construction and Transfection

HA‐hResistin, HA‐hResistinK2R, HA‐hResistin K19R, and HA‐hResistin K2/19R plasmids were synthesized from Hangzhou Guannan Biotechnology Co., Ltd. (Hangzhou, China). Flag‐OTUD6A‐, Flag‐OTUD6A C152A, Flag‐GFP‐△NT, Flag‐GFP‐△OTU, Flag‐△CT, Myc‐Ub WT, Myc‐Ub‐K48, and Myc‐Ub‐K63 plasmids were kindly provided by Dr. Guang Liang's research group at Wenzhou Medical University (Wenzhou, China) [15]. Plasmids were transiently transfected into Beas‐2B cells with lipofectamine3000 reagents (Invitrogen, Carlsbad, CA) according to the manufacturer's instructions.

### 4.4 Human Samples

A total of 2 lung biopsy samples were obtained from asthma patients with bronchially confirmed asthma who underwent pulmonary nodule resection (postoperatively diagnosed as benign nodules) at the Second Affiliated Hospital and Yuying Children's Hospital of Wenzhou Medical University (Wenzhou, China). Additionally, 2 adjacent normal lung tissue samples were from patients consecutively admitted during the same period for resection of pulmonary nodules (also confirmed as benign by histopathology). Informed consent was obtained from all participants, and the study was approved by the Clinical research ethics committee of the Second Affiliated Hospital and Yuying Children's Hospital of Wenzhou Medical University (Approval document: #2023‐K‐224‐0). Basic information concerning the patients is summarized in Figure S1A. Ki67 immunohistochemical staining showed no obvious of focal high proliferative activity (Figure S1B).

### 4.5 Animal Models

OTUD6A knockout mice (C57BL/6J background, Otud6a^−/−^) were kindly provided by Dr. Guang Liang's research group at Wenzhou Medical University (Wenzhou, China). All mice were housed under specific‐pathogen free (SPF) conditions with controlled humidity (50% ± 5%) and temperature (22 ± 2°C), under a 12‐h light/dark cycle. Prior to experimental procedures, animals were acclimatized to the laboratory environment for a minimum of one week. All protocols involving animal use were approved by the Institutional Animal Policy and Welfare Committee of Wenzhou Medical University (Approval No. wydw2022‐0938).

Before the formal asthma modeling experiments, we conducted a preliminary evaluation of the baseline status of Otud6a^−/−^ and wild‐type control mice. No significant differences were observed between the two groups in terms of serum IgE levels (Figure S11A), IL‐5 and IL‐13 levels in BALF (Figure S11B,C), and lung homogenates (Figure S11D,E). These results suggests that OTUD6A deficiency alone does not alter the baseline immune status. Therefore, in order to focus resources on exploring the role of OTUD6A in the pathogenic process of asthma, the primary experimental design in this study focused on comparing the disease phenotype differences between wild‐type and Otud6a^−/−^ mice after allergen treatment, without setting up a control group for the Otud6a^−/−^ at the baseline.

### 4.6 Transduction of Adeno‐Associated Virus (AAV) Transduction

To achieve lung‐specific OTUD6A knockdown, fifteen six‐week‐old C57BL/6 mice were intratracheally instilled with 1 × 10^11^ vector genomes of AAV6 expressing an OTUD6A core promoter‐driven construct (AAV6‐Otud6a). The AAV6 serotype was selected for its high tropism for murine lung tissues [44]. The asthma model was established three weeks post‐transduction to allow for sufficient transgene expression and protein knockdown (data not shown).

### 4.7 HDM‐Induced Acute Asthma Model (HDM‐AAM)

HDM‐induced acute asthma model (HDM‐AAM) was established based on a previously reported protocol with modifications to better suit our investigative focus [45]. Ten female C57BL/6 wild‐type (WT) and five Otud6a^−/−^ mice (6‐8‐weeks old) were randomly divided into three groups: wild‐type control (WT‐CON), wild‐type HDM‐exposed model (WT‐HDM‐AAM), and Otud6a^−/−^ HDM‐exposed model (Otud6a^−/−^‐HDM‐AAM). On day 0, the WT‐HDM‐AAM and Otud6a^−/−^‐HDM‐AAM groups were sensitized via intratracheal instillation of 100 µg HDM in 25 µL phosphate‐buffered saline (PBS). From days 14 to 17, mice received daily challenges with 10 µg HDM (25 µL PBS). The WT‐CON group received PBS alone at equivalent time points. Pulmonary function was assessed 24 h after the final challenge (day 18), followed by euthanasia and collection of serum, BALF, and lung tissue.

### 4.8 HDM‐Induced Chronic Asthma Model (HDM‐CAM)

For chronic asthma induction with associated airway remodeling, a model was established as previously described [20, 46]. Briefly, ten female C57BL/6 wild‐type and five Otud6a^−/−^ mice (6‐8‐weeks) old were randomly divided into three groups: WT‐CON, WT‐HDM‐CAM, and Otud6a^−/−^‐HDM‐CAM. Mice in the WT‐HDM‐CAM and Otud6a^−/−^‐HDM‐CAM groups received intratracheal instillations of 25 µg HDM (40 µL PBS) for 5 consecutive days per week, followed by a two‐day interval, repeating this cycle for five weeks. WT‐CON group mice received 40 µL sterile PBS. Pulmonary function was measured 24 h after the final challenge (day 33), followed by euthanasia and collection of serum, BALF, and lung tissue.

### 4.9 OVA‐Induced Allergic Asthma Model

The OVA‐induced allergic asthma model was generated using a well‐characterized two‐phase protocol [47]. Briefly, ten female C57BL/6 wild‐type and five Otud6a^−/−^ mice (6‐8‐weeks old) were randomly divided into three groups: WT‐CON, wild‐type OVA‐treated model (WT‐OVA), and Otud6a^−/−^ OVA‐treated model (Otud6a^−/−^‐OVA). Mice were sensitized via intraperitoneal injection of 20 µg OVA emulsified with 2 mg aluminum hydroxide in 200 µL PBS on days 0 and 14. From days 25 to 31, mice were subjected to daily 30‐min airway challenges with 1% OVA or PBS using a PARI TurboBOY N medical compressed air nebulizer. Euthanasia and sample collection were performed 24 h after the final challenge (day 32).

### 4.10 IL‐33‐Induced Airway Inflammation Model

To examine the expression of OTUD6A in innate immune‐mediated airway inflammation, we employed a modified IL‐33‐driven model based on established protocols [48, 49]. In our adaptation, six female C57BL/6 wild‐type mice (6‐8‐weeks old) were randomly divided into two groups: control group (CON) and IL‐33 model group (IL‐33). Mice in the model group received intratracheal instillations of 0.5 µg rmIL‐33 (25 µL PBS) on day 0, day 1, day 2, and day 3. WT‐CON group mice received 25 µL sterile PBS. Euthanasia and sample collection were performed 24 h after the final challenge (day 4).

### 4.11 ALT‐Induced Airway Inflammation Model

Airway inflammation was also induced using ALT, a clinically relevant fungal allergen associated with asthma [50]. Thirteen female C57BL/6 wild‐type mice (6‐8‐weeks old) were randomly divided into two groups: control group (CON, n = 5) and ALT model group (ALT, n = 8). Mice in the model group received intratracheal instillations of 5 µg Alternaria alternata extracts (25 µL PBS) on day 0, day 1, day 2, and day 3. WT‐CON group mice received 25 µL sterile PBS. Euthanasia and sample collection were performed 24 h after the final challenge (day 4).

### 4.12 AHR Assessment

AHR was assessed using the FlexiVent system (SCIREQ, Canada) in anesthetized and tracheostomized mice [47]. Following stabilization, baseline measurements of lung resistance (Rn), Respiratory system resistance (Rrs), and Respiratory system elastance (Ers) were recorded. Mice were sequentially challenged with aerosolized saline (baseline), followed by incremental methacholine doses (3.125, 6.25, 12.5, 25, and 50 mg/mL). Rn was continuously monitored, while Rrs and Ers were computed for each dose and expressed as a percentage change from baseline.

### 4.13 Histopathological Study

The middle lobe of the right lung was fixed in 4% paraformaldehyde, embedded in paraffin, and sectioned at 4 µm thickness for histopathological examination. H&E staining was performed to evaluate inflammatory cell infiltration and bronchial epithelial thickness, with quantitative morphometric analysis conducted using ImageJ software (National Institutes of Health, MD, USA). Masson's Trichrome Staining was employed to assess collagen deposition, while PAS staining was employed to assess goblet cell hyperplasia and mucus secretion. All images were acquired under light microscopy, and staining protocols followed established laboratory standards

### 4.14 Measurement of Cytokines and IgE

ELISA was performed to quantify IL‐5 and IL‐13 from lung homogenate and BALF, IgE in serum, IL‐25, IL‐33, and TSLP in BALF and medium, as well as hResistin in medium following manufacturer protocols. Briefly, after blocking the plate, 100 µL of samples were added to an ELISA plate coated with monoclonal capture antibodies and incubated at room temperature for 2 h. Following five washes with PBST (PBS + 0.1% Tween‐20), horseradish peroxidase (HRP)‐conjugated detection antibodies were added and incubated for 1 h. Tetramethylbenzidine (TMB) substrate was used for color development, and the reaction was terminated with 2M H_2_SO_4_. Absorbance was measured at wavelength of 450 nm and 570 nm using a SpectraMax iD3 (Molecular Devices, CA, USA). TGF‐β1 levels in serum were examined by ELISA kits using similar methodology. Standard curves were generated using recombinant protein standards to determine analyte concentrations.

### 4.15 Real‐Time Quantitative Polymerase Chain Reaction (RT‐qPCR)

Total RNA was extracted from lung tissues or cultured cells using TRIzol reagent (Thermo Fisher, CA, USA) and quantified spectrophotometrically by measuring absorbance at 260 and 280 nm. Complementary DNA (cDNA) synthesis was performed with HiScript III RT SuperMix (Vazyme, Nanjing, China) according to the manufacturer's protocol. Quantitative PCR amplification was carried out using SYBR qPCR Master Mix (Vazyme, Nanjing, China) on a QuantStudio 3 Real‐Time PCR System (Thermo Fisher, CA, USA). Gene‐specific primer sequences are listed in Table S1. The relative gene expression levels were normalized to β‐actin. The data presented sets the average of the model group to 100.

### 4.16 Immunohistochemistry Staining

Immunohistochemistry staining was performed according to our previous reported publication [51]. Formalin‐fixed, paraffin‐embedded lung tissue sections (4 µm) were deparaffinized in xylene and rehydrated through a graded ethanol series. Antigen retrieval was performed using a pressure cooker in 10 mm sodium citrate buffer (pH 6.0). Endogenous peroxidase activity was quenched with 3% hydrogen peroxide, followed by blocking in 1% bovine serum albumin (BSA) for 30 min. Sections were incubated overnight at 4°C with primary antibodies against OTUD6A (rabbit monoclonal, 1:400), mRELMα (mouse monoclonal, 1:400), or Ki67 (mouse monoclonal, 1:1000). After washing, sections were incubated with appropriate secondary antibodies at 37°C for 60 min. Immunoreactivity was visualized using 3,3′‐diaminobenzidine (DAB) as the chromogen, followed by counterstaining with hematoxylin. Images were acquired with a light microscope (Nikon, Tokyo, Japan), and quantitative analysis was performed using Image J software (National Institutes of Health, MD, USA).

### 4.17 Immunofluorescence Staining

Immunofluorescence staining was performed according to a previous report [52]. Lung tissue sections or transfected BEAS‐2B cells were fixed in 4% paraformaldehyde for 15 min, then labeled with primary antibodies overnight at 4°C. Fluorophore‐conjugated secondary antibodies were applied for 1 h at 37°C, and nuclei were counterstained with DAPI. Nuclei were visualized through DAPI staining. Fluorescence images were captured using an Olympus microscope equipped with DP2‐BSW software (Olympus, Tokyo, Japan). The primary antibodies were listed as follows: OTUD6A (1:400), hResistin (1:200), E‐Cadherin (1:400), HA‐tag (1:100), Flag‐tag (1:1000), and Calnexin (1:500). Specificity was confirmed by negative control experiments omitting the primary antibody.

### 4.18 Western Blot

Protein lysates from lung tissue (100 µg) or cells (30 µg) were subjected to 10% sodium dodecyl sulfate polyacrylamide gel electrophoresis and transferred to polyvinylidene fluoride membranes (Bio‐Rad Laboratories Inc., CA, USA). Membranes were blocked in 5% non‐fat milk in tris‐buffered saline containing 0.05% Tween 20 for 1.5 h at room temperature, followed by overnight incubation at 4°C with primary antibodies. After washing, membranes were incubated with HRP‐conjugated secondary antibodies. Protein bands were visualized using enhanced chemiluminescence reagents (Bio‐Rad Laboratories Inc., CA, USA), and densitometric analysis was performed using Image J software (National Institutes of Health, MD, USA).

### 4.19 Wound Healing Assay

Wound healing assay was performed as previously reported [53]. BEAS‐2B in the logarithmic growth phase are seeded on 6‐well culture plates overnight. The next day, streak vertically with a 200 µL pipette tip and rinse three times with PBS to remove the cells that have been crossed out. Cells were transfected with sihResistin or siNC for 48 h, and then transfected with Flag‐OTUD6A or Flag‐NC for an additional 24 h. Acquire scratch width images of each well at 0 and 24 h using an inverted microscope (Nikon, Japan) and analyze using ImageJ software.

### 4.20 Co‐Immunoprecipitation (Co‐IP)

Co‐IP assay was performed as previously reported [54]. Transfected cells or lung tissues were lysed in ice‐cold lysis buffer (Cat# P0013, Beyotime, Shanghai, China) supplemented with protease inhibitor (Cat# HY‐K0010, MCE, Shanghai, China). Lysates were sonicated and centrifuged at 12 000 × g for 15 min at 4°C. A fraction of the supernatant was retained as input, while the remaining was incubated overnight with anti‐HA or anti‐Flag antibodies at 4°C with gentle rotation. Immune complexes were captured using Protein A/G agarose beads, washed extensively with PBS, and eluted with 5x FD DualColor Protein Loading Buffer (Cat# FD006, Fdbio Science, Hangzhou, China) at 100°C. Precipitated proteins were analyzed by western blotting.

### 4.21 Cycloheximide Chase Assay

The CHX chase assay was conducted based on a previously reported protocol with modifications [55]. To assess protein stability, BEAS‐2B cells were transiently transfected with Flag‐OTUD6A, its catalytically inactive mutant (Flag‐OTUD6A‐C152A), or an empty vector control, using Lipofectamine 3000 transfection reagent. Twenty‐four hours post‐transfection, cells were treated with CHX (100 µg/mL) to inhibit de novo protein synthesis and harvested at 0, 2, 4, 6 h after CHX administration. Cell lysates were subsequently subjected to Western blot assay to quantify hResistin protein levels.

### 4.22 RNA‐Sequencing and Transcriptomic Analysis

Total RNA was extracted from freshly isolated murine lung tissues and subjected to high‐throughput RNA sequencing (RNA‐seq) at Lianchuan Bio Technologies (Hangzhou, China). Paired‐end sequencing (2 × 150 bp) was performed on an Illumina Novaseq 6000 platform (Illumina, CA, USA). Raw sequencing data were processed and annotated using established bioinformatics pipelines by LC Bio Technologies, with subsequent comparative transcriptomic analyses performed on the dataset deposited in the Gene Expression Omnibus (GEO; accession number GSE269435).

### 4.23 TMT‐Based Quantitative Proteomics

Global proteomic profiling of lung tissue samples was conducted using tandem mass tag (TMT)‐labeled mass spectrometry Protein samples were digested with trypsin, labeled with TMT reagents, and analyzed by liquid chromatography‐tandem mass spectrometry (LC‐MS/MS) at Lianchuan Bio Technologies (Hangzhou, China). Raw MS/MS data were processed using MaxQuant (version 2.1.4.0) with the following parameters: Type: Reporter ion MS2: TMT6plex, TMT10plex, TMT16plex or TMT18plex; enzyme: Trypsin/P; maximum missed cleavages: 2; fixed modification: carbamidomethyl (C); variable modifications: oxidation (M) and acetyl (protein N‐term); precursor mass tolerance: 20 ppm; fragment mass tolerance: 0.05 Da. Match‐between‐runs and second peptide search was enabled. Proteins were identified against the Uniprot database, with false discovery rate (FDR) threshold set at 1% for both peptide spectrum matches and protein‐level identifications. Reverse and contaminant entries were excluded from downstream analyses.

### 4.24 Statistical Analysis

Data were analyzed using GraphPad Prism 8.0 software. Values are expressed as the mean±standard error of the mean (SEM). Statistical significance was determined using unpaired two‐tailed Student's t test or one‐way Analysis of Variance (ANOVA) followed by Dunn's post hoc test. p‐value < 0.05 was considered significant, denoted as *.

## Author Contributions

Conceptualization by Hui Zhang, Yali Zhang, and Weixi Zhang; Methodology by Yali Zhang and Chengguang Zhao; Validation by Weiting Pan, Xinru Xi, Wei Dai, Tingfang Xiao, Yeqing Chen, and Xuanyu Chen; Data Curation, Weiting Pan, Xinru Xi, and Chengguang Zhao; Writing – Original Draft Preparation by Weiting Pan and Xinru Xi; Clinical Sample collection by Xiangting Ge; Writing – Review & Editing by Yali Zhang, Chengguang Zhao, and Weixi Zhang; Funding Acquisition by Hui Zhang, Yali Zhang and Weixi Zhang.

## Conflicts of Interest

The authors declare no conflicts of interest.

## Supporting information




**Supporting File**: advs73907‐sup‐0001‐SuppMat.docx.

## Data Availability

The data that support the findings of this study are available in the supplementary material of this article.

## References

[1] C. Porsbjerg , E. Melén , L. Lehtimäki , and D. Shaw , “Asthma,” Lancet 401, no. 10379 (2023): 858–873, 10.1016/s0140-6736(22)02125-0. 36682372

[2] H. Hammad and B. N. Lambrecht , “The Basic Immunology of Asthma,” Cell 184, no. 6 (2021): 1469–1485, 10.1016/j.cell.2021.02.016. 33711259

[3] I. H. Heijink , V. N. S. Kuchibhotla , M. P. Roffel , et al., “Epithelial Cell Dysfunction, a Major Driver of Asthma Development,” Allergy 75, no. 8 (2020): 1902–1917, 10.1111/all.14421. 32460363 PMC7496351

[4] J. M. Drazen and J. J. Fredberg , “Epithelial Cells Crowded out in Asthma,” Science 384, no. 6691 (2024): 30–31, 10.1126/science.ado4514. 38574157

[5] D. P. Potaczek , S. Miethe , V. Schindler , F. Alhamdan , and H. Garn , “Role of Airway Epithelial Cells in the Development of Different Asthma Phenotypes,” Cellular Signalling 69 (2020): 109523, 10.1016/j.cellsig.2019.109523. 31904412

[6] D. G. Rayner , D. M. Ferri , G. H. Guyatt , et al., “Inhaled Reliever Therapies for Asthma: A Systematic Review and Meta‐Analysis,” Jama 333, no. 2 (2025): 143–152, 10.1001/jama.2024.22700. 39465893 PMC11519786

[7] R. Wang and G. Wang , “Protein Modification and Autophagy Activation,” Advances in Experimental Medicine and Biology 1206 (2019): 237–259, 10.1007/978-981-15-0602-4_12. 31776989

[8] M. J. Schuijs , M. A. Willart , K. Vergote , et al., “Farm Dust and Endotoxin Protect Against Allergy Through A20 Induction in Lung Epithelial Cells,” Science 349, no. 6252 (2015): 1106–1110, 10.1126/science.aac6623. 26339029

[9] H. Vroman , D. van Uden , I. M. Bergen , et al., “Tnfaip3 expression in Pulmonary Conventional Type 1 Langerin‐expressing Dendritic Cells Regulates T Helper 2‐mediated Airway Inflammation in Mice,” Allergy 75, no. 10 (2020): 2587–2598, 10.1111/all.14334. 32329078 PMC7687104

[10] X. Hou , F. Zhu , Y. Ni , et al., “USP4 is Pathogenic in Allergic Airway Inflammation by Inhibiting Regulatory T Cell Response,” Life Sciences 281 (2021): 119720, 10.1016/j.lfs.2021.119720. 34144056

[11] J. Kumagai , M. Kiuchi , K. Kokubo , et al., “The USP7‐STAT3‐granzyme‐Par‐1 Axis Regulates Allergic Inflammation by Promoting Differentiation of IL‐5‐producing Th2 Cells,” The Proceedings of the National Academy of Sciences (PNAS) 120, no. 49 (2023): 2302903120, 10.1073/pnas.2302903120. PMC10710068 38015852

[12] S. Chen , F. Yun , Y. Yao , et al., “USP38 Critically Promotes Asthmatic Pathogenesis by Stabilizing JunB Protein,” Journal of Experimental Medicine 215, no. 11 (2018): 2850–2867, 10.1084/jem.20172026. 30224386 PMC6219735

[13] L. Shi , J. Liu , Y. Peng , et al., “Deubiquitinase OTUD6A Promotes Proliferation of Cancer Cells via Regulating Drp1 Stability and Mitochondrial Fission,” Molecular Oncology 14, no. 12 (2020): 3169–3183, 10.1002/1878-0261.12825. 33070427 PMC7718948

[14] Y. Zhao , X. Huang , D. Zhu , et al., “Deubiquitinase OTUD6A Promotes Breast Cancer Progression by Increasing TopBP1 Stability and Rendering Tumor Cells Resistant to DNA‐damaging Therapy,” Cell Death and Differentiation 29, no. 12 (2022): 2531–2544, 10.1038/s41418-022-01036-6. 35768646 PMC9751275

[15] X. Liu , Y. Fang , X. Lv , et al., “Deubiquitinase OTUD6A in Macrophages Promotes Intestinal Inflammation and Colitis via Deubiquitination of NLRP3,” Cell Death and Differentiation 30, no. 6 (2023): 1457–1471, 10.1038/s41418-023-01148-7. 36932155 PMC10244424

[16] Z. Fang , J. Han , L. Lin , et al., “Deubiquitinase OTUD6a Drives Cardiac Inflammation and Hypertrophy by Deubiquitination of STING,” Biochim Biophys Acta Mol Basis Dis 1870, no. 4 (2024): 167061, 10.1016/j.bbadis.2024.167061. 38342418

[17] X. Sun , S. Chen , Y. Zhao , et al., “OTUD6A in Tubular Epithelial Cells Mediates Angiotensin II‐induced Kidney Injury by Targeting STAT3,” American Journal of Physiology‐Cell Physiology 326, no. 2 (2024): C400–C413, 10.1152/ajpcell.00394.2023. 38105755

[18] Z. Li , G. Li , Y. Li , et al., “Deubiquitinase OTUD6A Regulates Innate Immune Response via Targeting UBC13,” Viruses 15, no. 8 (2023): 1761, 10.3390/v15081761. 37632103 PMC10458163

[19] P. W. Hellings and B. Steelant , “Epithelial Barriers in Allergy and Asthma,” Journal of Allergy and Clinical Immunology 145, no. 6 (2020): 1499–1509, 10.1016/j.jaci.2020.04.010. 32507228 PMC7270816

[20] T. Xu , Z. Wu , Q. Yuan , et al., “Proline Is Increased in Allergic Asthma and Promotes Airway Remodeling,” JCI Insight 8, no. 16 (2023): 167395, 10.1172/jci.insight.167395. PMC10543727 37432745

[21] J. Quan , D. Xie , Z. Li , et al., “Luteolin Alleviates Airway Remodeling in Asthma by Inhibiting the Epithelial‐Mesenchymal Transition via β‐Catenin Regulation,” Phytomedicine 135 (2024): 156090, 10.1016/j.phymed.2024.156090. 39393303

[22] M. Moghbeli , “PI3K/AKT Pathway as a Pivotal Regulator of Epithelial‐Mesenchymal Transition in Lung Tumor Cells,” Cancer cell international 24, no. 1 (2024): 165, 10.1186/s12935-024-03357-7. 38730433 PMC11084110

[23] M. G. Nair , K. J. Guild , and D. Artis , “Novel Effector Molecules in Type 2 Inflammation: Lessons Drawn From helminth Infection and Allergy,” Journal of Immunology 177, no. 3 (2006): 1393–1399, 10.4049/jimmunol.177.3.1393. PMC1780267 16849442

[24] R. Z. Yang , Q. Huang , A. Xu , et al., “Comparative Studies of Resistin Expression and Phylogenomics in Human and Mouse,” Biochemical and Biophysical Research Communications 310, no. 3 (2003): 927–935, 10.1016/j.bbrc.2003.09.093. 14550293

[25] J. Cui , X. Liu , Q. Shang , et al., “Deubiquitination of CDC6 by OTUD6A Promotes Tumour Progression and Chemoresistance,” Molecular Cancer 23, no. 1 (2024): 86, 10.1186/s12943-024-01996-y. 38685067 PMC11057083

[26] L. R. Bonser and D. J. Erle , “The Airway Epithelium in Asthma,” Advances in Immunology 142 (2019): 1–34, 10.1016/bs.ai.2019.05.001. 31296301

[27] A. Akenroye , J. A. Boyce , and H. Kita , “Targeting Alarmins in Asthma: From Bench to Clinic,” Journal of Allergy and Clinical Immunology 155, no. 4 (2025): 1133–1148, 10.1016/j.jaci.2025.01.017. 39855362 PMC12555011

[28] G. Varricchi , C. E. Brightling , C. Grainge , B. N. Lambrecht , and P. Chanez , “Airway Remodelling in Asthma and the Epithelium: On the Edge of a New Era,” European Respiratory Journal 63, no. 4 (2024): 2301619, 10.1183/13993003.01619-2023. 38609094 PMC11024394

[29] R. B. Werder , M. A. Ullah , M. M. Rahman , et al., “Targeting the P2Y(13) Receptor Suppresses IL‐33 and HMGB1 Release and Ameliorates Experimental Asthma,” American Journal of Respiratory and Critical Care Medicine 205, no. 3 (2022): 300–312, 10.1164/rccm.202009-3686OC. 34860143 PMC12042653

[30] L. Yuan , H. Liu , X. Du , et al., “Airway Epithelial ITGB4 Deficiency Induces Airway Remodeling in a Mouse Model,” Journal of Allergy and Clinical Immunology 151, no. 2 (2023): 431–446, 10.1016/j.jaci.2022.09.032. 36243221

[31] H. Chen , J. Liu , J. Zhang , et al., “USP22/BRD4 Mediated Hedgehog Pathway Activation Contributes to Airway Remodeling in Asthma,” International Immunopharmacology 153 (2025): 114538, 10.1016/j.intimp.2025.114538. 40132456

[32] Y. Yokoyama , T. Tamachi , A. Iwata , et al., “A20 (Tnfaip3) Expressed in CD4(+) T Cells Suppresses Th2 Cell‐Mediated Allergic Airway Inflammation in Mice,” Biochemical and Biophysical Research Communications 629 (2022): 47–53, 10.1016/j.bbrc.2022.08.097. 36099784

[33] H. Vroman , T. Das , I. M. Bergen , et al., “House Dust Mite‐Driven Neutrophilic Airway Inflammation in Mice With TNFAIP3‐Deficient Myeloid Cells Is IL‐17‐Independent,” Clinical and Experimental Allergy 48, no. 12 (2018): 1705–1714, 10.1111/cea.13262. 30171721

[34] Q. Lin and R. A. Johns , “Resistin Family Proteins in Pulmonary Diseases,” American Journal of Physiology‐Lung Cellular and Molecular Physiology 319, no. 3 (2020): L422–L434, 10.1152/ajplung.00040.2020. 32692581 PMC7518061

[35] S. Jiang , D. W. Park , J. M. Tadie , et al., “Human Resistin Promotes Neutrophil Proinflammatory Activation and Neutrophil Extracellular Trap Formation and Increases Severity of Acute Lung Injury,” Journal of Immunology 192, no. 10 (2014): 4795–4803, 10.4049/jimmunol.1302764. PMC4018664 24719460

[36] H. Zhang , Y. Zhu , Z. Liu , et al., “A Volatile from the Skin Microbiota of Flavivirus‐Infected Hosts Promotes Mosquito Attractiveness,” Cell 185, no. 14 (2022): 2510, 10.1016/j.cell.2022.05.016. 35777355

[37] Q. Lin , C. Fan , J. T. Skinner , et al., “RELMα Licenses Macrophages for Damage‐Associated Molecular Pattern Activation to Instigate Pulmonary Vascular Remodeling,” Journal of Immunology 203, no. 11 (2019): 2862–2871, 10.4049/jimmunol.1900535. PMC6864303 31611261

[38] C. Fan , Z. Fu , Q. Su , et al., “S100A11 Mediates Hypoxia‐Induced Mitogenic Factor (HIMF)‐induced Smooth Muscle Cell Migration, Vesicular Exocytosis, and Nuclear Activation,” Molecular & Cellular Proteomics 10, no. 3 (2011): M110.000901, 10.1074/mcp.M110.000901. PMC3047144 21139050

[39] J. Zhao , X. Jiao , J. Wu , et al., “FIZZ1 Promotes Airway Remodeling in Asthma Through the PTEN Signaling Pathway,” Inflammation 38, no. 4 (2015): 1464–1472, 10.1007/s10753-015-0121-5. 25655389

[40] M. Zhang , J. Lin , J. Zhang , et al., “Artesunate Inhibits Airway Remodeling in Asthma via the MAPK Signaling Pathway,” Frontiers in pharmacology 14 (2023): 1145188, 10.3389/fphar.2023.1145188. 36998616 PMC10043319

[41] J. C. Christianson , E. Jarosch , and T. Sommer , “Mechanisms of Substrate Processing During ER‐Associated Protein Degradation,” Nature Reviews Molecular Cell Biology 24, no. 11 (2023): 777–796, 10.1038/s41580-023-00633-8. 37528230

[42] L. Krshnan , M. L. van de Weijer , and P. Carvalho , “Endoplasmic Reticulum‐Associated Protein Degradation,” Cold Spring Harbor Perspectives in Biology 14, no. 12 (2022): a041247, 10.1101/cshperspect.a041247. 35940909 PMC9732900

[43] J. Bousquet , J. M. Anto , C. Bachert , et al., “Allergic Rhinitis,” Nature Reviews Disease Primers 6, no. 1 (2020): 95, 10.1038/s41572-020-00227-0. 33273461

[44] J. Wang , X. Lai , S. Yao , et al., “Nestin Promotes Pulmonary Fibrosis via Facilitating Recycling of TGF‐β Receptor I,” European Respiratory Journal 59, no. 5 (2022): 2003721, 10.1183/13993003.03721-2020. 34625478 PMC9068978

[45] M. A. Ullah , J. A. Revez , Z. Loh , et al., “Allergen‐Induced IL‐6 Trans‐Signaling Activates Γδ T Cells to Promote Type 2 and Type 17 Airway Inflammation,” Journal of Allergy and Clinical Immunology 136, no. 4 (2015): 1065–1073, 10.1016/j.jaci.2015.02.032. 25930193

[46] Z. Sun , N. Ji , Q. Ma , et al., “Epithelial‐Mesenchymal Transition in Asthma Airway Remodeling Is Regulated by the IL‐33/CD146 Axis,” Frontiers in immunology 11 (2020): 1598, 10.3389/fimmu.2020.01598. 32793232 PMC7387705

[47] C. Lu , B. Zhang , T. Xu , et al., “Piperlongumine Reduces Ovalbumin‑Induced Asthma and Airway Inflammation by Regulating Nuclear Factor‑κB Activation,” International Journal of Molecular Medicine 44, no. 5 (2019): 1855–1865, 10.3892/ijmm.2019.4322. 31485644 PMC6777695

[48] P. Shafiei‐Jahani , D. G. Helou , B. P. Hurrell , et al., “CD200‐CD200R Immune Checkpoint Engagement Regulates ILC2 Effector Function and Ameliorates Lung Inflammation in Asthma,” Nature Communications 12, no. 1 (2021): 2526, 10.1038/s41467-021-22832-7. PMC8100131 33953190

[49] L. She , G. D. Barrera , L. Yan , et al., “STING Activation in Alveolar Macrophages and Group 2 Innate Lymphoid Cells Suppresses IL‐33‐driven Type 2 Immunopathology,” JCI Insight 6, no. 3 (2021): 143509, 10.1172/jci.insight.143509. 33400692 PMC7934858

[50] V. D. Gandhi , J. Y. Cephus , A. E. Norlander , et al., “Androgen Receptor Signaling Promotes Treg Suppressive Function During Allergic Airway Inflammation,” Journal of Clinical Investigation 132, no. 4 (2022): 153397, 10.1172/jci153397. PMC8843736 35025767

[51] Y. Zhang , T. Xu , Z. Pan , et al., “Shikonin Inhibits Myeloid Differentiation Protein 2 to Prevent LPS‐induced Acute Lung Injury,” British Journal of Pharmacology 175, no. 5 (2018): 840–854, 10.1111/bph.14129. 29243243 PMC5811619

[52] Y. Wang , W. Luo , J. Han , et al., “MD2 Activation by Direct AGE Interaction Drives Inflammatory Diabetic Cardiomyopathy,” Nature Communications 11, no. 1 (2020): 2148, 10.1038/s41467-020-15978-3. PMC7195432 32358497

[53] Y. Chai , H. Xiang , Y. Ma , et al., “S1PR1 Suppresses Lung Adenocarcinoma Progression Through p‐STAT1/miR‐30c‐5 p/FOXA1 Pathway,” Journal of Experimental & Clinical Cancer Research 43, no. 1 (2024): 304, 10.1186/s13046-024-03230-5. 39551792 PMC11571582

[54] X. Xi , H. Chen , H. Ji , et al., “MD2 Mediates COPD Pathogenesis by Inducing Airway Inflammation and Ferroptosis Through the TLR4/MyD88 Pathway,” Biochimica et Biophysica Acta Molecular Basis of Disease 1872, no. 2 (2026): 168099, 10.1016/j.bbadis.2025.168099. 41177411

[55] Y. Zhao , J. Ruan , Z. Li , et al., “OTUB1 Inhibits Breast Cancer by Non‐Canonically Stabilizing CCN6,” Clinical and Translational Medicine 13, no. 8 (2023): 1385, 10.1002/ctm2.1385. PMC10444971 37608493

