# Supplementary material for: OTUD6A in Airway Epithelial Cells Exacerbates Allergic Asthma by Promoting Airway Inflammation and Airway Remodeling Through Deubiquitination of hResistin/mRELMα
Source: Adv Sci (Weinh). 2026 Jan 20;13(18):e16355. doi: 10.1002/advs.202516355 (PMC13042843; doi:10.1002/advs.202516355)
Supplement: Supplementary file 1 — Supporting File: advs73907‐sup‐0001‐SuppMat.docx. [file ADVS-13-e16355-s001.docx]

Supplementary Information

**OTUD6A in Airway Epithelial Cells Exacerbates Allergic Asthma by Promoting Airway Inflammation and Airway Remodeling through Deubiquitination of hResistin/mRELMα**

Weiting Pan^1,2,#^, Xinru Xi^2,#^, Wei Dai^1,#^, Tingfang Xiao^2,#^, Yeqing Chen^1^, Xuanyu Chen^2^, Xiangting Ge^1^, Chengguang Zhao^1,2^, Hui Zhang^1,3,^*, Yali Zhang^2,^*, Weixi Zhang^1,3,^*

^1^Department of Pediatric Allergy and Immunology, The Second Affiliated Hospital and Yuying Children’s Hospital of Wenzhou Medical University, Wenzhou, Zhejiang, 325027, China

^2^Chemical Biology Research Center, School of Pharmaceutical Sciences, Wenzhou Medical University, Wenzhou, Zhejiang, 325035, China

^3^Zhejiang Provincial Clinical Research Center for Pediatric Disease, Wenzhou, Zhejiang, 325027, China


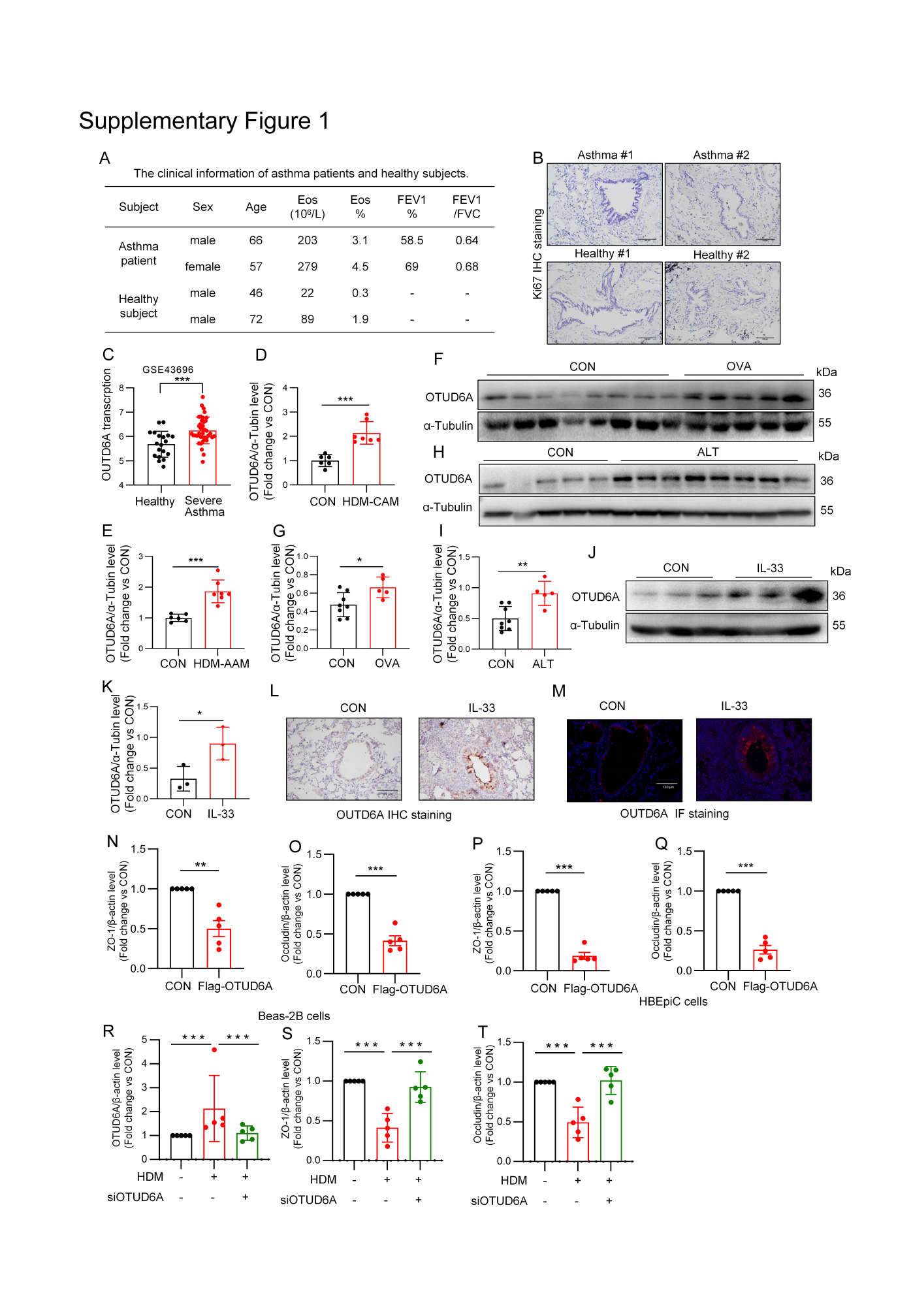


**Figure. S1 OTUD6A expression is upregulated in asthma patients and mouse.**

**A-B)** The clinical information of asthma patients and healthy subjects showed in Figure 1A, and analysis of Ki67 expression in airways. Scale bars: 100 μm**. C)** OTUD6A transcription in GSE43696 dataset. **D)** Quantification of immunoblots in Figure 1C (*n* = 6-7). **E)** Quantification of immunoblots in Figure 1D (*n* = 6-7). **F-I)** Western blot analysis and quantification of OTUD6A expression in lung tissue of OVA-induced asthma mice (F, G), ALT-treated mice (H, I) and IL-33-treated mice (J-K) (*n* = 3-7). **L-M)** Representative images of IHC and IF for OTUD6A on lung sections of IL-33-treated mice. Scale bars: 100 μm. **N-O)** Quantification of immunoblots in Figure 1L (*n* = 5). **P-Q)** Quantification of immunoblots in Figure 1N (*n* = 5). **R-T)** Quantification of immunoblots in Figure 1P (*n* = 5). Data are presented as mean ± SEM. *P* values determined by two-tailed unpaired t-test (**P* < 0.05, ***P* < 0.01, a****P* < 0.001).


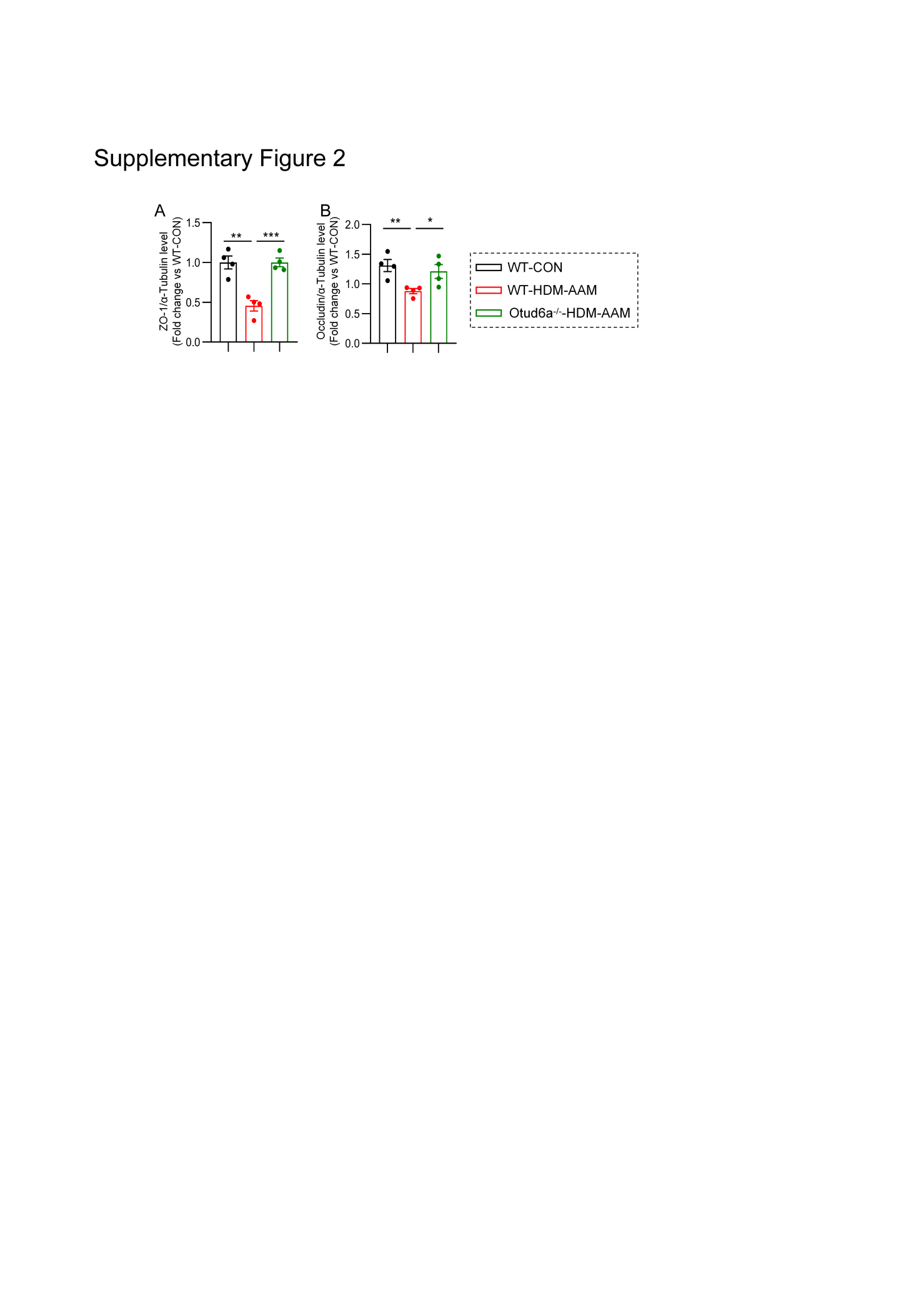


**Figure S2. OTUD6A knockout alleviates epithelial barrier disruption in HDM-induced acute asthma model.**

**A-B)** Quantification of immunoblots in Figure 2P (*n* = 5). Data are presented as mean ± SEM. *P* values determined by one-way ANOVA (**P* < 0.05, ***P* < 0.01, ****P* < 0.001).

**
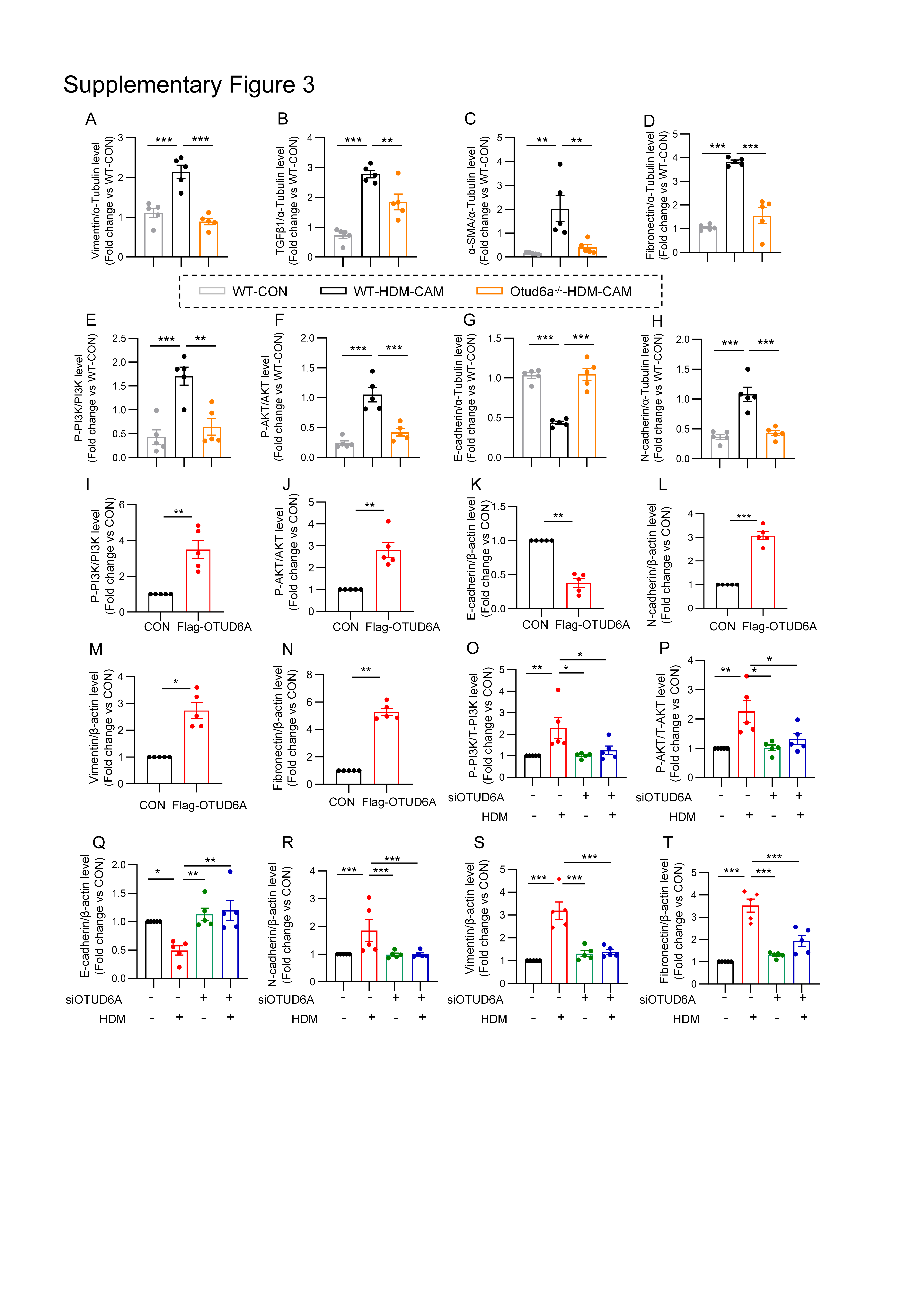
**

**Figure S3. OTUD6A promotes airway remodeling via PI3K/AKT-EMT signaling.**

**A-D)** Quantification of immunoblots in Figure 4D (*n* = 5). **E-H)** Quantification of immunoblots in Figure 4F (*n* = 5). **I-N)** Quantification of immunoblots in Figure 4G (*n* = 5). **O-P)** Quantification of immunoblots in Figure 4H (*n* = 5). **Q-T)** Quantification of immunoblots in Figure 4I (*n* = 5). Data are presented as mean ± SEM. *P* values determined by one-way ANOVA (**P* < 0.05, ***P* < 0.01, ****P* < 0.001)


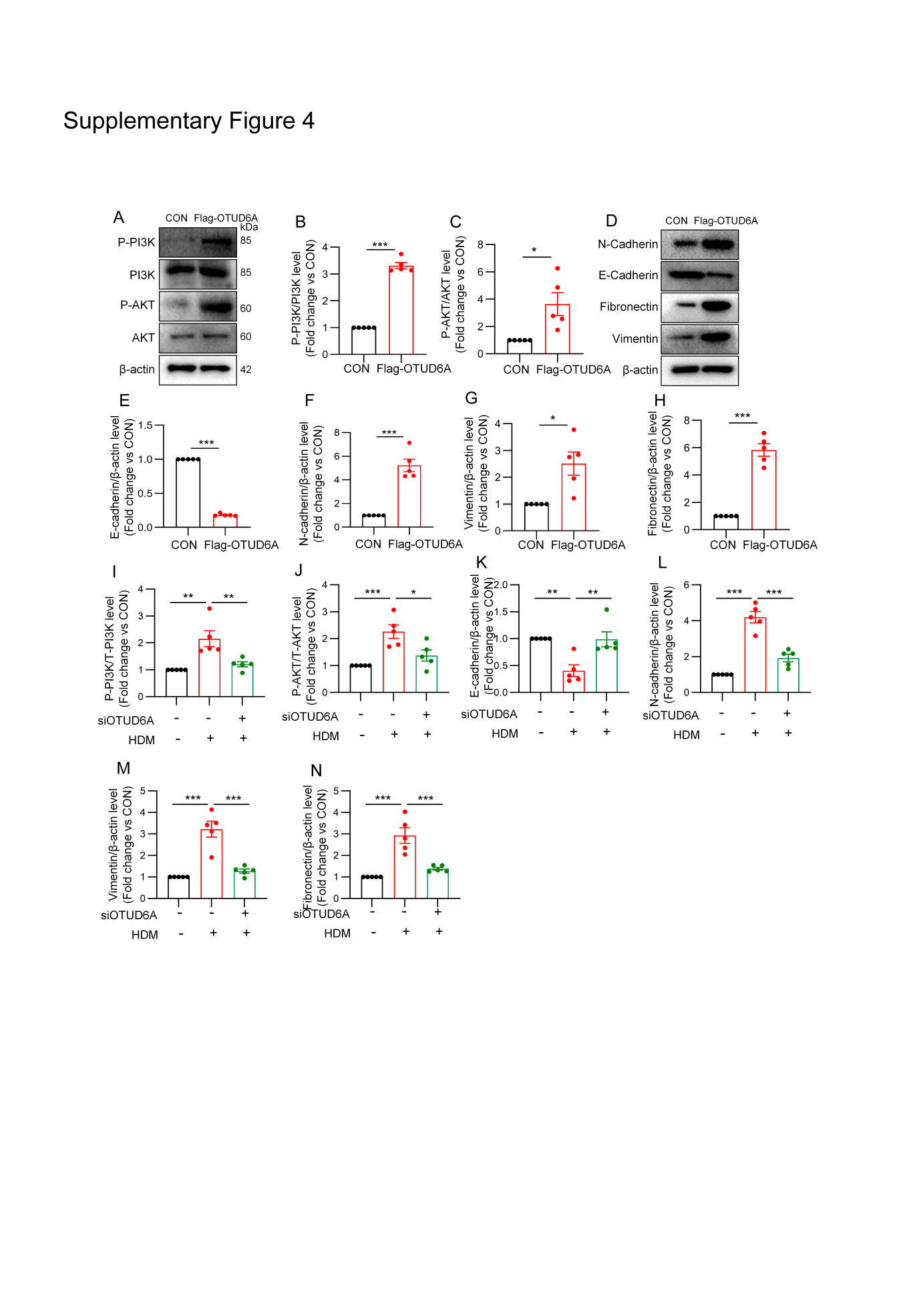


**Figure S4. OTUD6A regulates PI3K/AKT signaling pathway and EMT process in HBEpiC cells.**

**A-H)** Western blot analysis and quantification of PI3K/AKT (A-C) and EMT markers (D-H) in HBEpiC cells transfected with Flag-OTUD6A and control vector for 24 h (*n* = 5). **I)** Quantification of immunoblots in Figure 4K (*n* = 5). **J)** Quantification of immunoblots in Figure 4L (*n* = 5). **K-L)** Quantification of immunoblots in Figure 4M (*n* = 5). **M-N)** Quantification of immunoblots in Figure 4N (*n* = 5). Data are presented as mean ± SEM. *P* values determined by one-way ANOVA (**P* < 0.05, **P < 0.01, ***P < 0.001).


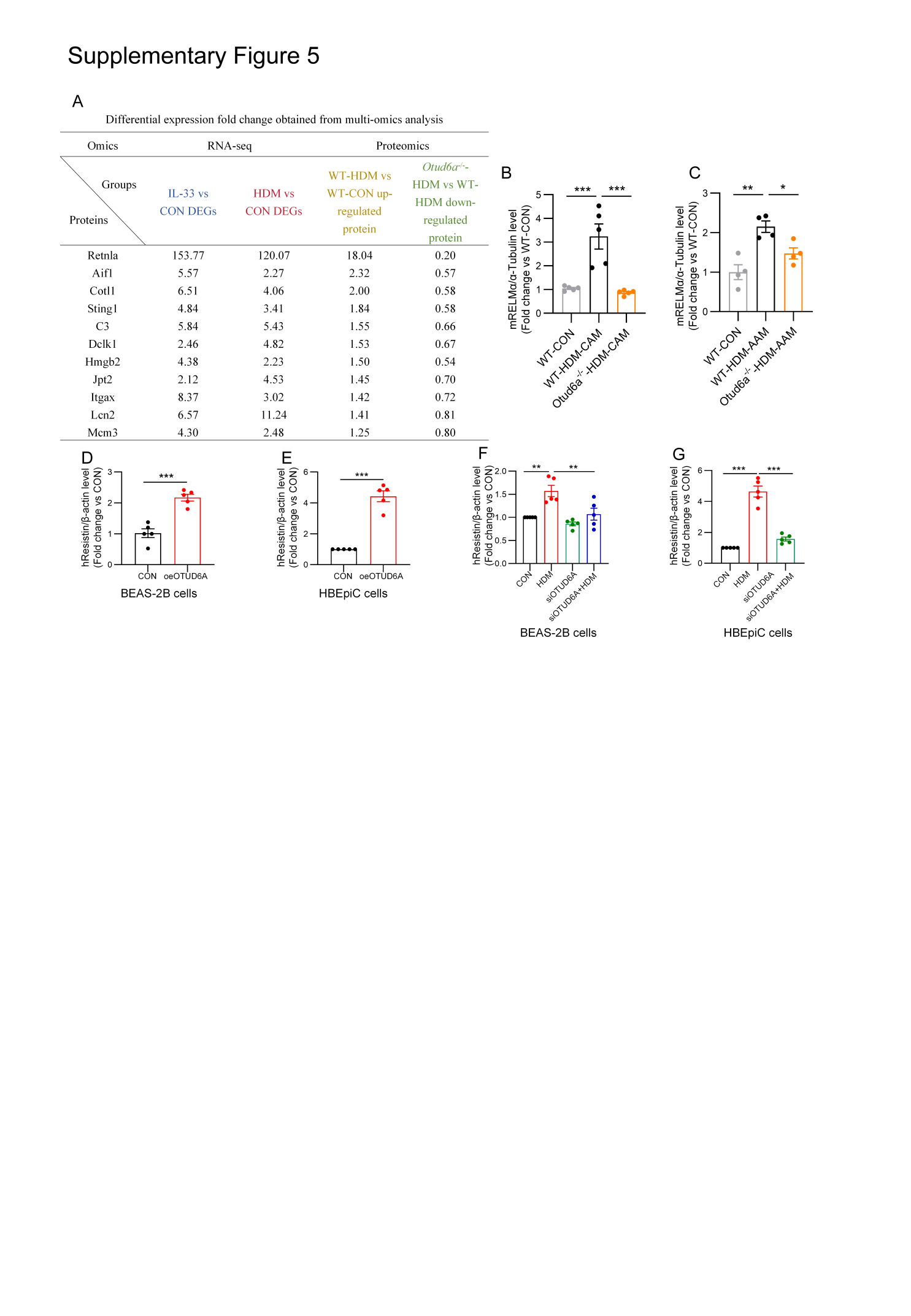


**Figure S5. HResistin/mRELMα is a potential substrate for OTUD6A.**

**A)** The difference multiples of the 11 differentially expressed genes obtained from the intersection in Figure 4A in multi-omics analysis. **B)** Quantification of immunoblots in Figure 5B (*n* = 5). **C)** Quantification of immunoblots in Figure 5C (*n* = 4). **D)** Quantification of immunoblots in Figure 5H (*n* = 5). **E)** Quantification of immunoblots in Figure 5I (*n* = 5). **F)** Quantification of immunoblots in Figure 5J (*n* = 5). **G)** Quantification of immunoblots in Figure 5K (*n* = 5). Data are presented as mean ± SEM. *P* values determined by two-tailed unpaired t-test or one-way ANOVA (**P* < 0.05, ***P* < 0.01, ****P* < 0.001, ns: no significant).


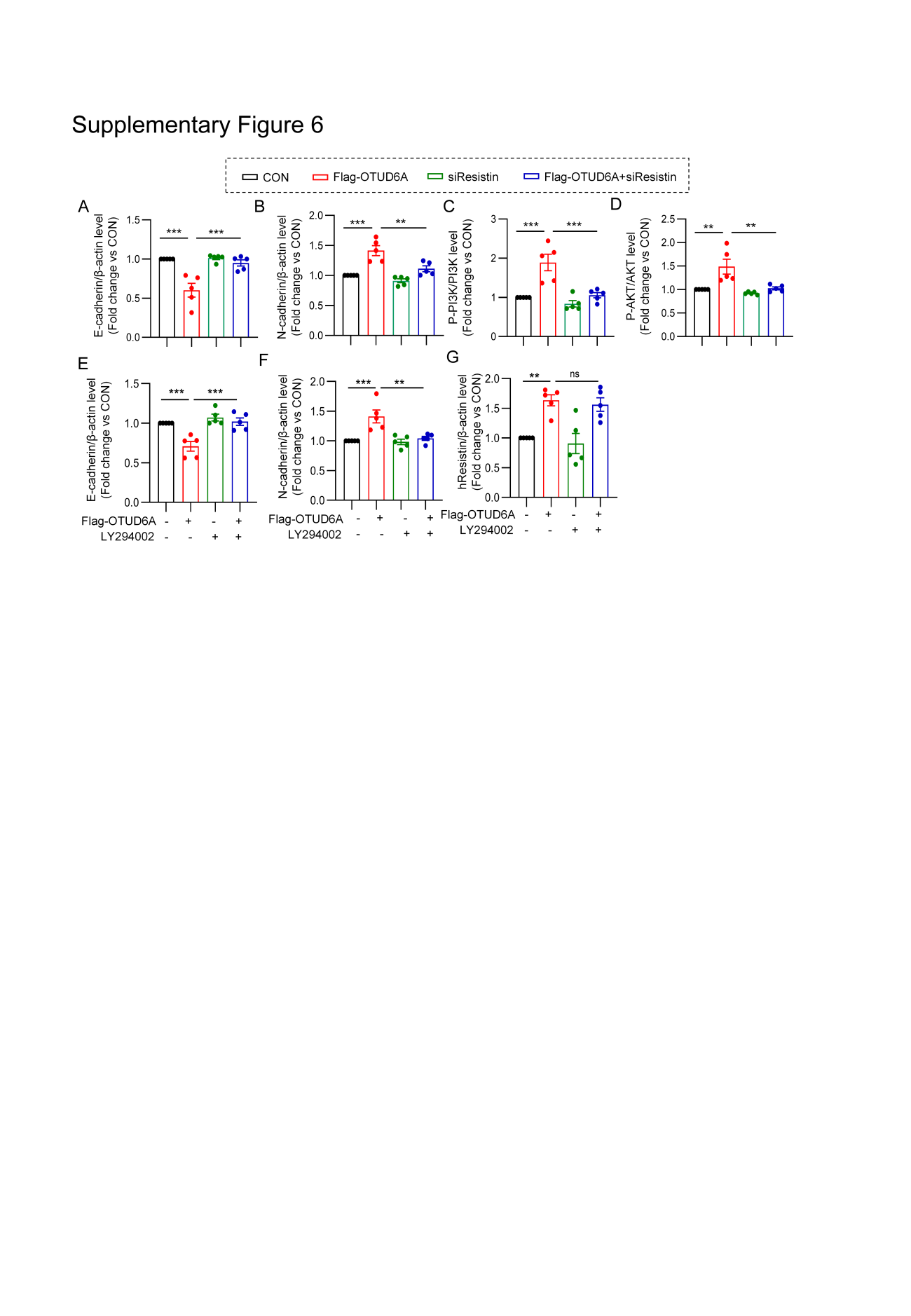


**Figure S6. OTUD6A regulates PI3K/AKT signaling pathway and EMT process via hResistin.**

**A-B)** Quantification of immunoblots in Figure 6D (*n* = 5). **C-D)** Quantification of immunoblots in Figure 6E (*n* = 5). **E-G)** Quantification of immunoblots in Figure 6H (*n* = 5). Data are presented as mean ± SEM. *P* values determined by one-way ANOVA (**P* < 0.05, ***P* < 0.01, ****P* < 0.001).


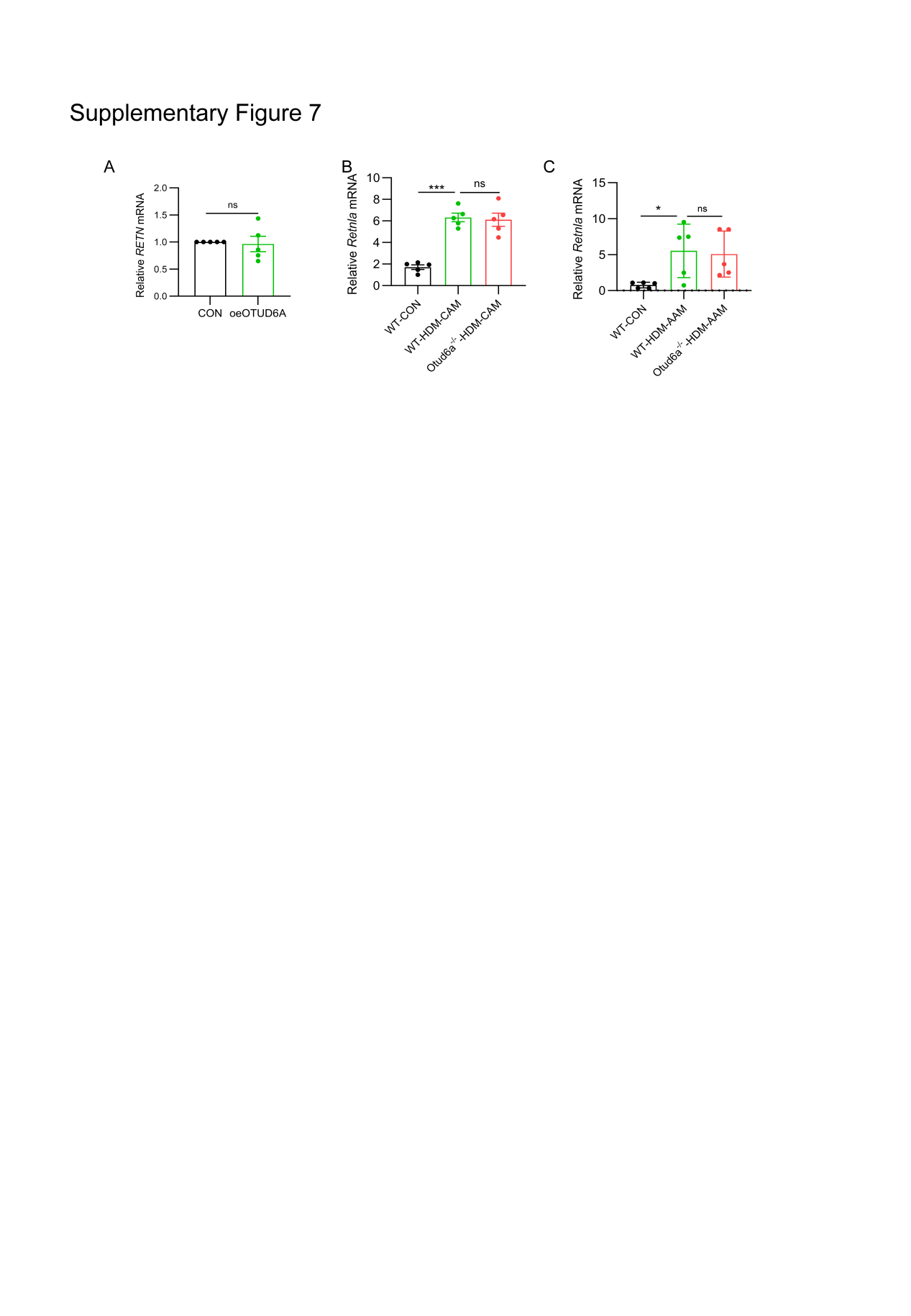


**Figure S7. OTUD6A has no effect on the mRNA levels of hResistin/mRELMα.**

**A)** Beas-2B cells were transfected with Flag-OTUD6A and control vector for 24 h. RT-qPCR analysis of RETN mRNA level (*n* = 5). **B-C)** RT-qPCR analysis of *Retnla* mRNA in HDM-induced chronic asthma model (B) and HDM-induced acute asthma model (C) (*n* = 5). Data are presented as mean ± SEM. *P* values determined by one-way ANOVA (**P* < 0.05, ***P* < 0.01, ****P* < 0.001, ns: no significant).


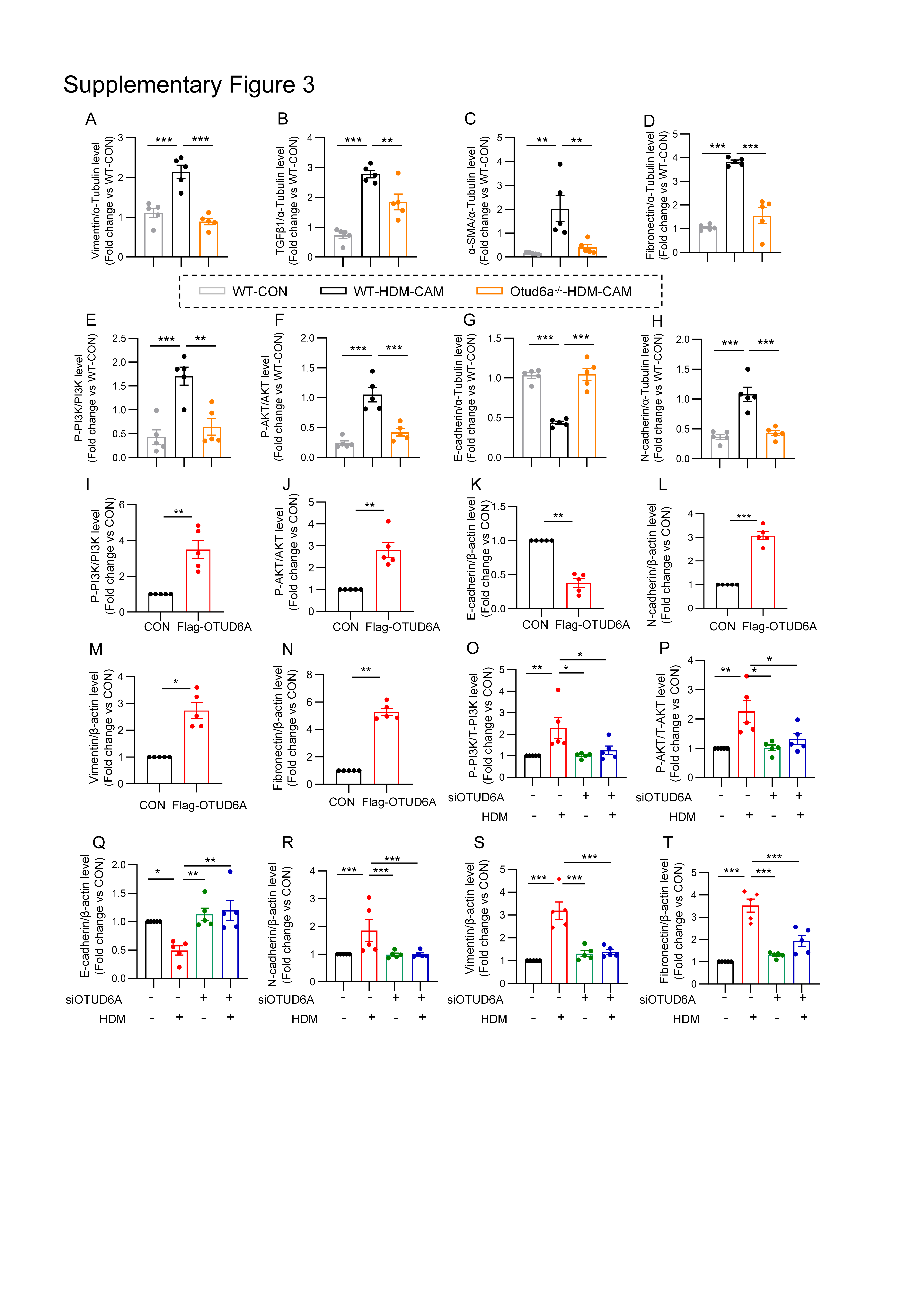


**Figure S8. Lung-specific OTUDU6A knockdown alleviates HDM-induced asthma.**

**A-B)** Quantification of immunoblots in Figure 9B (*n* = 5). **C-D)** Mice were stimulated wit h different concentrations of methacholine (0, 3.125, 6.25, 12.5, 25, 50 mg/mL) to evaluate their Rrs (C) and Ers (D) (*n* = 5). **E-F)** IL-5 (E) and IL-13 (F) levels in lung homogenates measured by ELISA (*n* = 5). **G)** RT-qPCR analysis of *Il25*, *Il33* and *Tslp* mRNA in HDM-induced chronic asthma model (*n* = 5). **H)** Fibrosis score using Masson trichrome staining in Figure 8O (*n* = 5). **I-L)** Quantification of immunoblots in Figure 9Q (*n* = 5). **M-N)** Quantification of immunoblots in Figure 9R (*n* = 5). **P-O)** Quantification of immunoblots in Figure 9S (*n* = 5). Data are presented as mean ± SEM. *P* values determined by one-way ANOVA (**P* < 0.05, ***P* < 0.01, ****P* < 0.001).


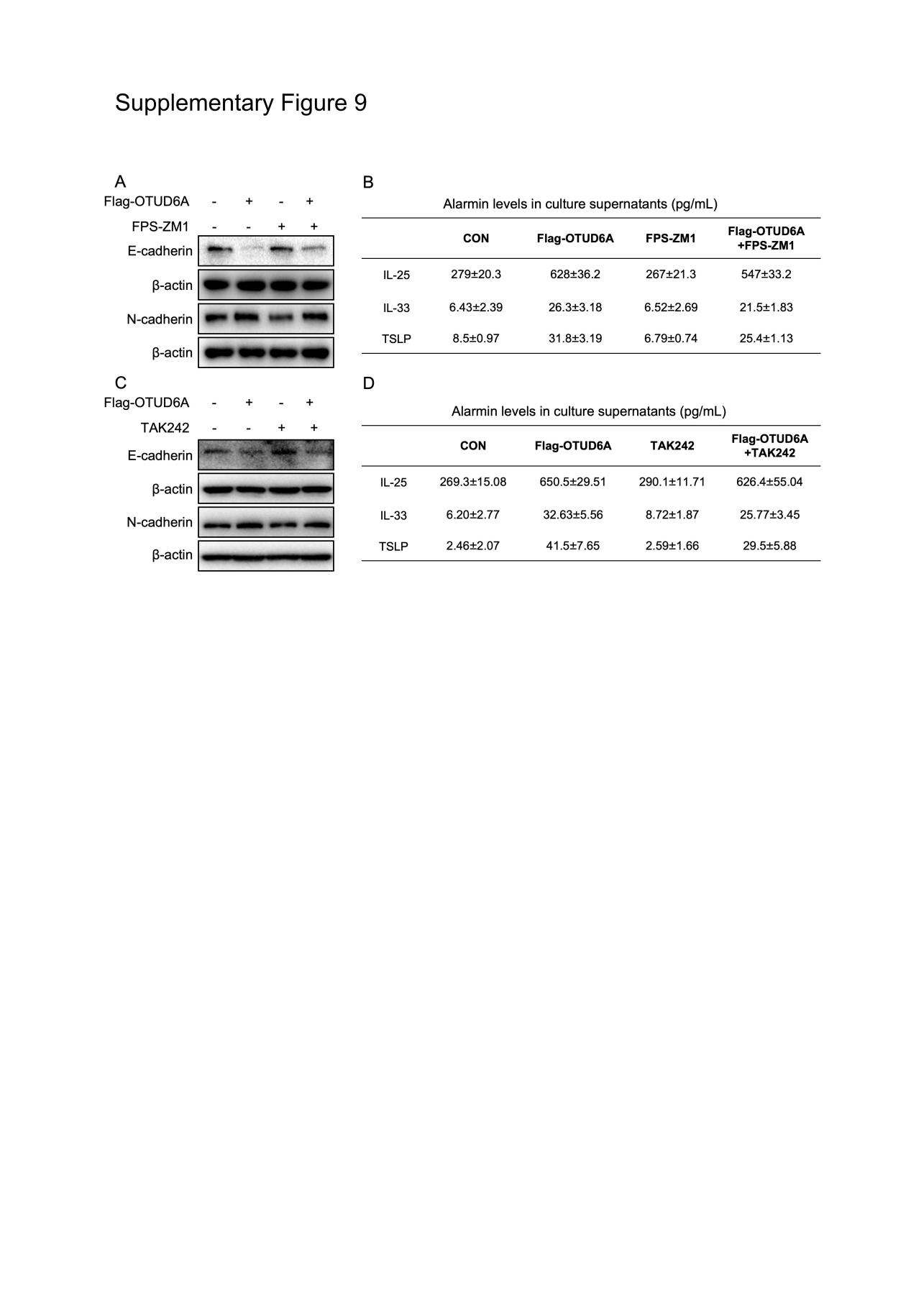


**Figure S9. RAGE and TLR4 are not involved in OTUD6A/hResistin mediated process.**

Beas-2B cells were pretreated with FPS-ZM1 (10 μM) or TAK242 (2.5 μM) and transfected with Flag-OTUD6A or control vector for 24 h. **A, C)** Western blot analysis of E-cadherin and N-cadherin. **B, D)** IL-25, IL-33 and TSLP levels in culture supernatants measured by ELISA.


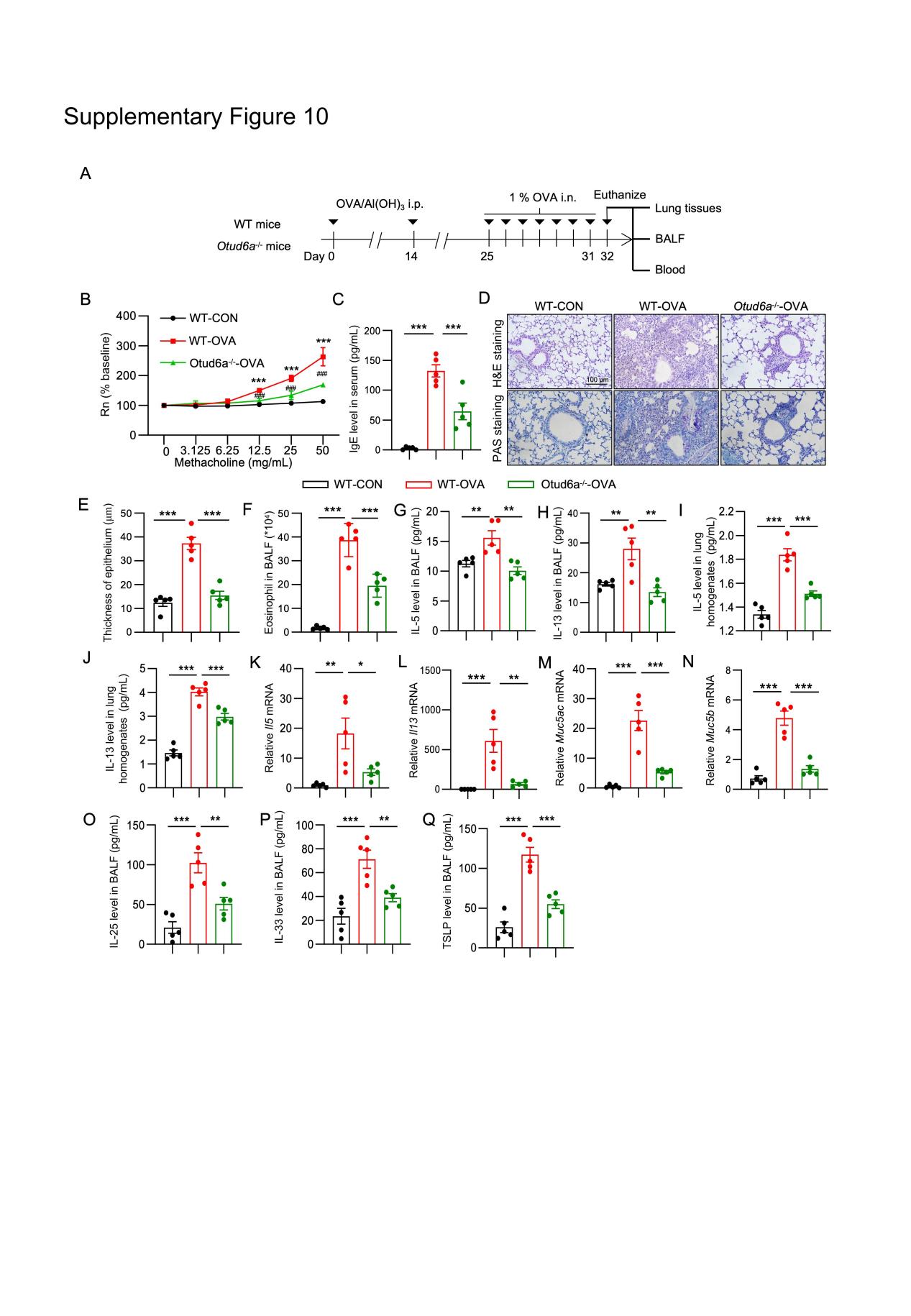


**Supplementary Figure S10. OTUD6A knockout alleviates OVA-induced asthma.**

**A)** Schematic diagram depicting the procedure of OVA-induced asthma model. **B)** Mice were stimulated with different concentrations of acetylcholine (0, 3.125, 6.25, 12.5, 25, 50 mg/mL) to evaluate their airway hyperresponsiveness. **C)** Serum IgE levels were analyzed by ELISA. **D)** Representative images of H&E and PAS of lung tissue. Scale bars: 100 μm. **E)** Quantification of bronchial epithelial thickness in mice. **F)** BALF eosinophil counts were determined by Wright-Giemsa staining. **G-J)** IL-5 and IL-13 levels in BALF (G, H) and lung homogenates (I, J) were measured by ELISA. **K-N)** RT-qPCR analysis of *Il5*, *Il13*, *Mua5ac*, and *Muc5b* mRNA in lung tissues. **O-Q)** IL-25, IL-33, and TSLP levels in culture supernatants measured by ELISA. *n* = 5 for each group. Data are presented as mean ± SEM. *P* values determined by one-way ANOVA (**P* < 0.05, ***P* < 0.01, ****P* < 0.001).


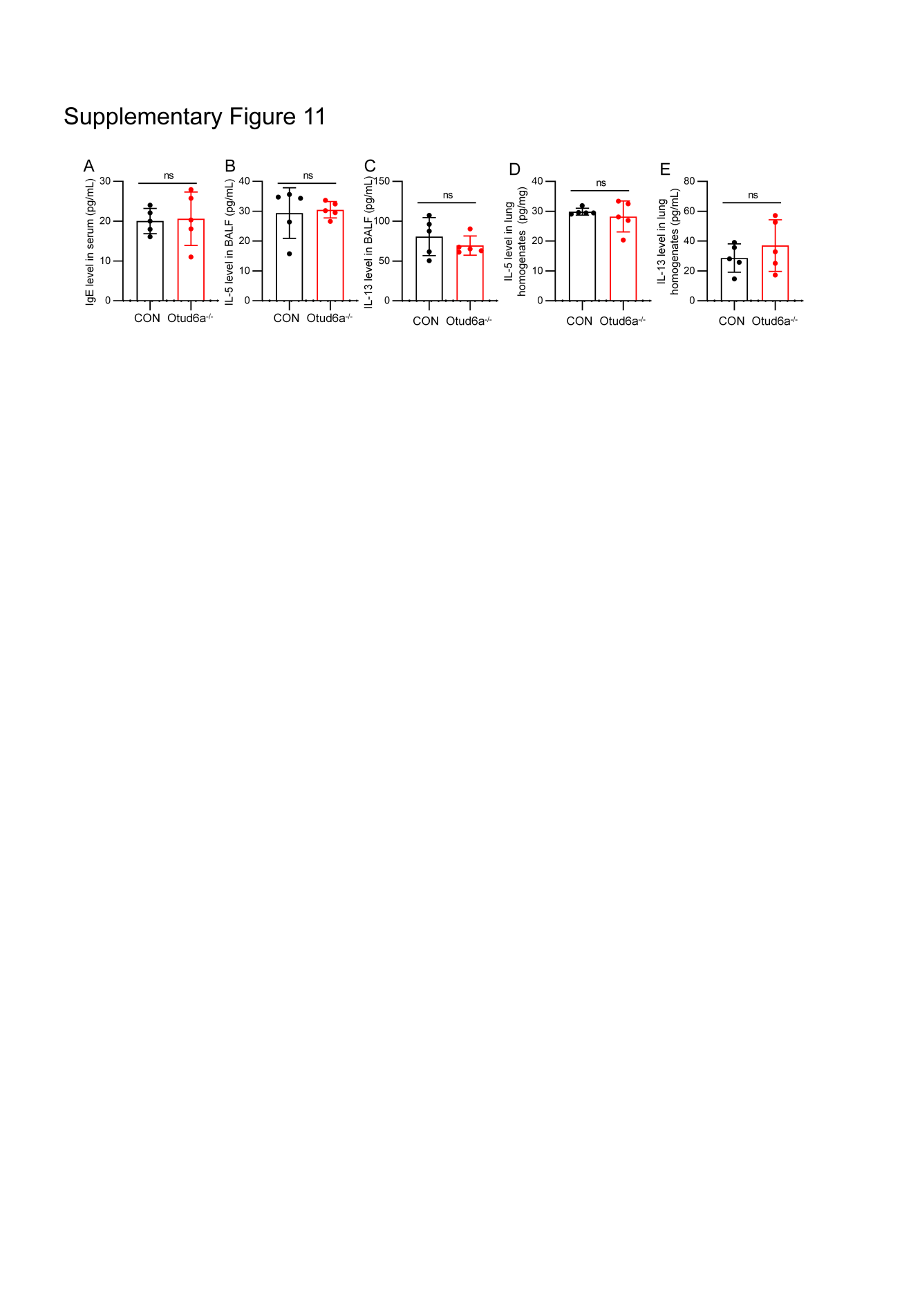


**Supplementary Figure S11. OTUD6A knockout does not affect the basal immune condition.**

**A)** Serum IgE levels. **B-E)** IL-5 and IL-13 levels in BALF **(B, D)** and lung homogenates **(C, E)** measured by ELISA (*n* = 5). Data are presented as mean ± SEM. *P* values determined by two-tailed unpaired t-test (ns: no significant).

**Supplementary Table S1. Primer sequences for RT-qPCR assay.**

| Gene | Species | Forward Primer | Reverse Primer |
| --- | --- | --- | --- |
| *β-actin* | human | CCTGGCACCCAGCACAAT | GCCGATCCACACGGAGTACT |
| *RETN* | human | CCCACCGAGAGGGATGAAAG | CCAATGCTGCTTATTGCCCT |
| *IL25* | human | CCAAGTGGAGTGAGAAACTGGGATC | AGTGGCTGTAGGTGTGGGTTCC |
| *IL33* | human | GTGACGGTGTTGATGGTAAGAT | AGCTCCACAGAGTGTTCCTTG |
| *TSLP* | human | CGGCCACATTGCCTTACTGA | TAGCCTGGGCACCAGATAGC |
| *COL1A1* | human | GACCGATGGATTCCAGITCG | TGTGACTCGTGCAGCCATCG |
| *ACTA2* | human | AGACTTACGAGTTGCCTGATG | ATGAAGGATGGCTGGAACAG |
| *TGFB1* | human | AGTGGACATCAACGGGTTCAC | AAGTTGGCATGGTAGCCCTT |
| *β-actin* | mouse | CACTGTCGAGTCGCGTCC | TCATCCATGGCGAACTGGTG |
| *Muc5ac* | mouse | CTCTACTGACTGCACCAACACAT | CCTCCCATTACTGAGCAGGTG |
| *Muc5b* | mouse | GGTGTGGCCAGCAGAGAG | TCTGACTGTCTCCGGTGAGT |
| *Il4* | mouse | GCTCTCAACCCCCAGCTAGT | GCCGATGATCTCTCTCAAGTGAT |
| *Il5* | mouse | CAAGCAATGAGACGATGAGGC | AGCATTTCCACAGTACCCCC |
| *Il13* | mouse | GTTCTCTCACTGGCTCTGGG | CAGGGGAGTCTGGTCTTGTG |
| *Il25* | mouse | GTTCTCTCACTGGCTCTGGG | CAGGGGAGTCTGGTCTTGTG |
| *Il33* | mouse | TCCAACTCCAAGATTTCCCCG | CATGCAGTAGACATGGCAGAA |
| *Tslp* | mouse | ACGGATGGGGCTAACTTACAA | AGTCCTCGATTTGCTCGAACT |
| *Retnla* | mouse | CAGCTGATGGTCCCAGTGAAT | CAGTGGAGGGATAGTTAGCTGG |
